# Sodium and Its Impact on Outcome After Aneurysmal Subarachnoid Hemorrhage in Patients With and Without Delayed Cerebral Ischemia

**DOI:** 10.1097/CCM.0000000000006182

**Published:** 2024-01-11

**Authors:** Homeyra Labib, Maud A. Tjerkstra, Bert A. Coert, René Post, W. Peter Vandertop, Dagmar Verbaan, Marcella C.A. Müller

**Affiliations:** 1 Department of Neurosurgery, Amsterdam UMC location University of Amsterdam, Neurosurgery, Amsterdam, The Netherlands.; 2 Amsterdam Neurosciences, Neurovascular Disorders, Amsterdam, The Netherlands.; 3 Department of Intensive Care, Amsterdam UMC location University of Amsterdam, Amsterdam, The Netherlands.

**Keywords:** aneurismal subarachnoid hemorrhage, cerebral ischemia, critical care, stroke, subarachnoid hemorrhage, sodium, prognosis

## Abstract

**OBJECTIVES:**

To perform a detailed examination of sodium levels, hyponatremia and sodium fluctuations, and their association with delayed cerebral ischemia (DCI) and poor outcome after aneurysmal subarachnoid hemorrhage (aSAH).

**DESIGN:**

An observational cohort study from a prospective SAH Registry.

**SETTING:**

Tertiary referral center focused on SAH treatment in the Amsterdam metropolitan area.

**PATIENTS:**

A total of 964 adult patients with confirmed aSAH were included between 2011 and 2021.

**INTERVENTIONS:**

None.

**MEASUREMENTS AND MAIN RESULTS:**

A total of 277 (29%) developed DCI. Hyponatremia occurred significantly more often in DCI patients compared with no-DCI patients (77% vs. 48%). Sodium levels, hyponatremia, hypernatremia, and sodium fluctuations did not predict DCI. However, higher sodium levels were significantly associated with poor outcome in DCI patients (DCI onset –7, DCI +0, +1, +2, +4, +5, +8, +9 d), and in no-DCI patients (postbleed day 6–10 and 12–14). Also, hypernatremia and greater sodium fluctuations were significantly associated with poor outcome in both DCI and no-DCI patients.

**CONCLUSIONS:**

Sodium levels, hyponatremia, and sodium fluctuations were not associated with the occurrence of DCI. However, higher sodium levels, hypernatremia, and greater sodium fluctuations were associated with poor outcome after aSAH irrespective of the presence of DCI. Therefore, sodium levels, even with mild changes in levels, warrant close attention.

**KEY POINTS**

**Question:** To determine the relation between sodium levels, occurrence of hyponatremia, hypernatremia, fluctuations of sodium levels and delayed cerebral ischemia (DCI), and poor outcome after aneurysmal subarachnoid hemorrhage (aSAH).

**Findings:** In this single-tertiary center cohort study, daily sodium levels, hyponatremia, hypernatremia, and fluctuations of sodium levels were not predictive of DCI. Higher sodium levels, hypernatremia, and greater fluctuations of sodium levels were significantly associated with poor outcome in both patients with and without DCI.

**Meaning:** Sodium levels, even with mild changes, appear to have a negative impact on clinical outcome after aSAH.

Aneurysmal subarachnoid hemorrhage (aSAH) carries a high risk of morbidity and mortality in relatively young patients (1–4). A great contributor to this poor outcome is delayed cerebral ischemia (DCI), occurring in ±33% patients (5–7). If risk factors for developing DCI could be identified in advance, this might enable prophylactic interventions (8, 9).

To predict DCI, countless biomarkers related to the multifactorial pathophysiology of DCI have been investigated (10, 11). Essentially, any event that decreases cerebral perfusion pressure (CPP) and cerebral blood flow (CBF) could contribute to the development of DCI (12–14). As volemic status is closely related to CPP and CBF, inducing hypervolemia was recommended in the past with the assumption of increasing CPP and CBF, but studies failed to show beneficial effects (15). However, studies did show that fluid restriction was associated with a higher occurrence rate of DCI (16–18). Therefore, current guidelines recommend euvolemia and fluid management guided by fluid balance (8, 9, 19). Because euvolemia is not clearly defined and subject to interpretation, this has often been translated to maintaining a positive fluid balance in clinical practice. However, recent studies showed that higher fluid intake was also associated with DCI and fluid restriction without compromising cardiac preload and CBF was possible (20–22), which conflicts with the knowledge on which guidelines are based (16–18).

Nonetheless, the emphasis in guidelines still lies on volemic status with deviations from normality being associated with DCI, therefore factors such as sodium may be useful in identifying patients at risk of DCI. Because dysregulations of sodium are associated with changes in volemic status (23, 24), and hyponatremia (30–56%) is one of the most common electrolyte disturbances after aSAH, monitoring sodium meticulously could be important (8, 25–29). However, the current literature shows conflicting evidence regarding sodium (disorders), fluctuations, and DCI (16, 17, 30–45), with most of the studies not taking the day of DCI onset into account, while this is crucial in determining a causal relation. Likewise, the relation between sodium disorders and outcome shows conflicting evidence (31, 35, 37, 43, 44, 46–53).

The main objective is to examine the relation between sodium, dysnatremia, sodium fluctuations, and DCI and poor outcome at 6 months after aSAH, while taking the day of DCI onset into account.

## MATERIALS AND METHODS

### Patient Population

A single-center cohort study from a prospective SAH Registry was performed. This registry included consecutive SAH patients admitted to the Amsterdam UMC, a tertiary SAH referral center in the Amsterdam metropolitan area. Adult patients (≥ 18 yr) admitted between December 2011 and December 2021 were included if SAH was confirmed by noncontrast computer tomography/lumbar puncture due to a ruptured aneurysm confirmed by CT-angiography and/or digital subtraction angiography, with at least one sodium measurement in blood in the first 14 days after ictus. Patients with non-aSAH, death within 3 days after ictus and unavailable DCI status were excluded. This study was conducted in accordance with the Helsinki Declaration. Ethical approval by the institutional review board of the Amsterdam UMC was not necessary as this study did not fall under the board’s guidelines as human subjects research.

### Data Collection

The following data were used from the prospective SAH Registry: age, sex, hypertension, hypercholesterolemia, cardiovascular disease, World Federation of Neurosurgical Societies scale (WFNS) at treatment center admission (54), modified Fisher grade (55), aneurysm location, treatment modality, DCI defined according to Vergouwen et al (56), day of DCI onset, complications during admission (rebleeding, hydrocephalus, meningitis, and seizures), and clinical outcome at 6 months assessed with the modified Rankin Scale (mRS) (57–59). Sodium levels were extracted retrospectively from the electronic patient records. Definitions of the variables are described in **Supplementary materials** (http://links.lww.com/CCM/H480).

### Sodium

As DCI occurs mainly between days 3–14 after ictus, sodium levels during the first 14 days after ictus were collected if available (postbleed day [PBD] 0–14) (60). If sodium was measured multiple times a day, the lowest sodium level was used for analysis. Hyponatremia was defined as a sodium level less than 135 mmol/L. Hypernatremia was defined as a sodium level greater than 145 mmol/L. Sodium fluctuation was defined as the difference between the highest and lowest absolute sodium level per mmol/L.

In DCI patients, sodium levels were centered around the day of DCI onset. The occurrence of dysnatremia and sodium fluctuations in DCI patients were evaluated in three distinct time intervals; between ictus and DCI onset (before DCI onset), between DCI onset and PBD 14 (after DCI onset), and during admission (PBD 0–14). This was done to gain a better understanding in whether hyponatremia and sodium fluctuations precede or follow DCI onset.

In no-DCI patients, dysnatremia and sodium fluctuations were evaluated during admission (PBD 0–14).

### Patient Management

Patients were treated according to our institutional protocol based on (inter)national guidelines (8, 19). A detailed description is available in the Supplementary materials (http://links.lww.com/CCM/H480).

### Statistical Analysis

The first main outcome was the occurrence of DCI during admission. The second main outcome was poor outcome (mRS 4–6) at 6 months after aSAH. The normality of the data was tested using the Shapiro-Wilk test (W ≤ 0.90 was considered skewed). Data were presented as absolute numbers with percentages (%), median with interquartile range or mean with sd. The chi-square, Fisher exact-test, Mann-Whitney *U*, or independent *T*-test were used accordingly for analyzing group differences.

Sodium levels and fluctuations were graphically visualized and compared during PBD 0–14 in subgroups based on DCI status, WFNS (WFNS 1–2 and 3–5) and location of ruptured aneurysm (anterior communicating artery [ACOM] and no ACOM). For DCI, we compared the sodium levels and fluctuations before DCI onset and after DCI onset of DCI patients with sodium levels and fluctuations during admission of no-DCI patients.

The predictive ability of sodium-related variables for DCI was assessed with logistic regression analyses by calculating odds ratios (OR) and 95% CIs. Daily sodium levels before DCI onset in DCI patients and daily sodium levels during admission in no-DCI patients were used as the independent variable. Adjusted OR with 95% CI were calculated using multivariate logistic regression analyses with adjustments for known predictors of DCI (age, sex, WFNS, and modified Fisher grade) (61). Additionally, to evaluate whether the presence of DCI is predictive of sodium-related variables, logistic or linear regression analyses were repeated accordingly with DCI as the independent variable. In linear regression analyses, unadjusted and adjusted regression coefficients with 95% CI (Beta and aBeta, respectively) were calculated. All analyses were repeated for poor outcome versus good outcome. IBM SPSS Statistics (version 28) and GraphPad Prism (version 9.1.0) were used for statistical analyses and graphic presentations, respectively. Statistical significance was set at two-sided *p* value of less than 0.05.

## RESULTS

The cohort comprised of 964 patients with a mean age 57 (sd 13) of whom 682 (71%) were female, 316 (33%) presented with WFNS grades 4–5 and 585 (61%) had modified Fisher 4. Hyponatremia occurred in 545 patients (57%) during admission. Clinical outcome at 6 months was available for 895 patients (93%) of whom 295 (33%) had poor outcome (**Table S1** and **Fig. S1**, http://links.lww.com/CCM/H480).

DCI occurred in 277 patients (29%) at mean day 7 (sd 4) after ictus. DCI patients were more often female (*p* = 0.041) and had higher modified Fisher grades (*p* < 0.001). Hydrocephalus, meningitis, seizures, poor outcome occurred significantly more, and hypernatremia less in DCI patients than no-DCI patients (*p* < 0.001 and *p* = 0.021, respectively).

### Sodium and DCI

**Figure 1.**
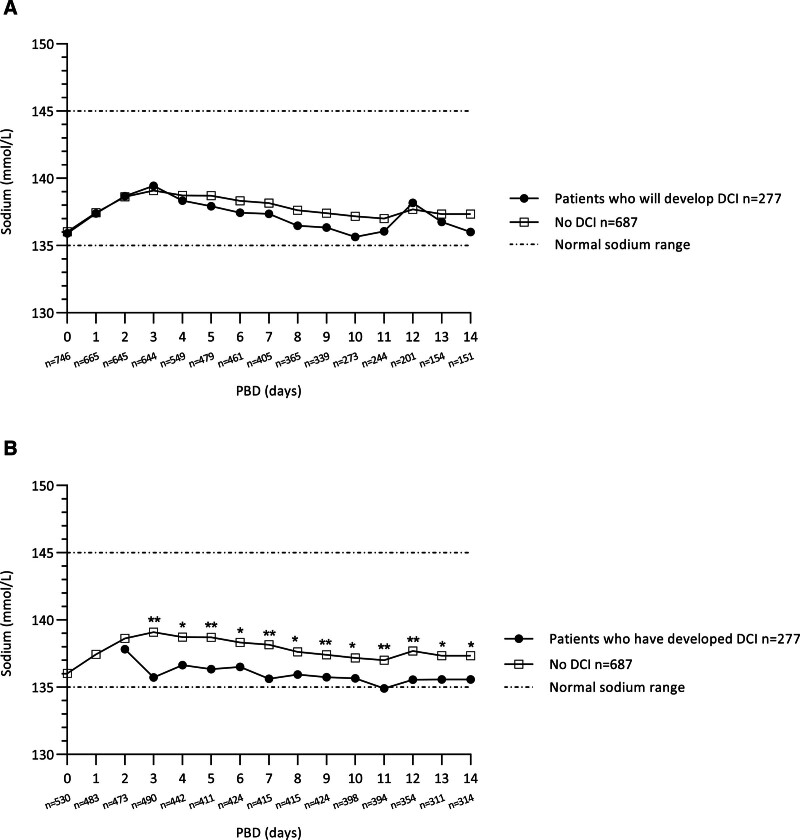
Daily sodium levels in patients with and without delayed cerebral ischemia (DCI). **A**, In patients with DCI, only sodium levels before the onset of DCI were used for analyzing group differences. For example, if a patient developed DCI on postbleed day (PBD) 4, only sodium levels on PBD 0, 1, 2, and 3 were included in the analysis. **B**, In patients with DCI, only sodium levels after the onset of DCI were used for analyzing group differences. For example, if a patient developed DCI on PBD 4, only sodium levels on PBD 5, 6, and so forth were included in the analysis. Days are presented as PBD with PBD 0 as the day of aneurysmal subarachnoid hemorrhage (aSAH) ictus. The number of available sodium measurements (*n* = *x*) per day are noted under the *x*-axis. *p < 0.05, **p < 0.001.

Before DCI onset, sodium levels in patients who would develop DCI were not significantly different from patients who did not develop DCI (**Fig. 1*A***; and **Table S2*A***, http://links.lww.com/CCM/H480). Multivariate logistic regression analysis showed that daily sodium levels were not predictive of the development of DCI (**Table S4**, http://links.lww.com/CCM/H480). After the onset of DCI, sodium levels were significantly lower in DCI patients than no-DCI patients (**Fig. 1*B***; and **Table S2*B***, http://links.lww.com/CCM/H480). Multivariate linear regression analysis showed that the presence of DCI was significantly predictive of lower sodium levels on PBD 3–14 (**Table S5**, http://links.lww.com/CCM/H480).

During admission, hyponatremia occurred significantly more in DCI patients (77%) than no-DCI patients (48%) (Table S1, http://links.lww.com/CCM/H480). Within the DCI group, an episode of hyponatremia was observed in 54% and 59% of the patients before and after DCI onset, respectively (Table S1, http://links.lww.com/CCM/H480). Hyponatremia nor hypernatremia were predictive of the development of DCI in multivariate logistic regression analysis (**Table 1**). The presence of DCI was significantly predictive of the development of hyponatremia in multivariate logistic regression analysis (aOR 1.46; 95% CI, 1.07–1.98; *p* = 0.017) but not for hypernatremia (**Table S6**, http://links.lww.com/CCM/H480).

**TABLE 1.** The Relation Between Hyponatremia, Hypernatremia, Sodium Fluctuations, and Delayed Cerebral Ischemia After Aneurysmal Subarachnoid Hemorrhage

PBD	DCI	
	OR (95% CI)	aOR (95% CI)^a^
Hyponatremia before DCI onset or PBD 0–14^b^	1.24 (0.94–1.65)	1.16 (0.87–1.56)
Hypernatremia before DCI onset or PBD 0–14^c^	1.04 (0.69–1.58)	0.98 (0.63–1.51)
Sodium fluctuations before DCI onset or PBD 0–14^d^	1.00 (0.98–1.03)	0.99 (0.96–1.02)

aOR = adjusted odds ratio, DCI = delayed cerebral ischemia, OR = odds ratio, PBD = postbleed days, WFNS = World Federation of Neurosurgical Societies scale.

a Adjusted for age, sex, WFNS, and modified Fisher grade. For the multivariate logistic regression six patients were excluded due to unknown WFNS grade (*n* = 5) and unknown modified Fisher grade (*n* = 1).

b Univariate logistic regression analysis with hyponatremia as the independent variable. This analysis was carried out in 952 patients of whom 265 (28%) eventually developed DCI. Patients with DCI were scored as hyponatremia if they had a sodium level < 135 mmol/L before the onset of DCI. Patients without DCI were scored as hyponatremia if they had a sodium level < 135 mmol/L at any time point during PBD 0–14.

c Univariate logistic regression analysis with hypernatremia as the independent variable. This analysis was carried out in 952 patients of whom 265 (28%) eventually developed DCI. Patients with DCI were scored as hypernatremia if they had a sodium level > 145 mmol/L before the onset of DCI. Patients without DCI were scored as hypernatremia if they had a sodium level > 145 mmol/L at any time point during PBD 0–14.

d Univariate logistic regression analysis with sodium fluctuations as the independent variable. This analysis was carried out in 929 patients of whom 261 (28%) eventually developed DCI..

Before and after the onset of DCI, sodium fluctuations were not significantly different in DCI patients from no-DCI patients (**Fig. S2**, ***A*** and ***B***, http://links.lww.com/CCM/H480). Sodium fluctuations were not predictive of the development of DCI in multivariate logistic regression analysis (Table 1). The presence of DCI was significantly predictive of smaller sodium fluctuations in multivariate linear regression analysis (aBeta –1.25; 95% CI, –2.11 to –0.39; *p* = 0.005) (Table S6, http://links.lww.com/CCM/H480).

### Sodium and WFNS

Sodium levels were significantly lower on PBD 0–1 and higher on PBD 13–14 in WFNS 3–5 patients than WFNS 1–2 patients (**Fig. S3**, http://links.lww.com/CCM/H480).

Sodium fluctuations were significantly higher in WFNS 3–5 patients compared than WFNS 1–2 patients (**Fig. S4**, http://links.lww.com/CCM/H480).

### Sodium and ACOM Aneurysm

Sodium levels and fluctuations did not differ between ACOM and no ACOM patients (**Figs. S5** and **S6**, http://links.lww.com/CCM/H480).

### Sodium, Clinical Outcome, and DCI

**Figure 2.**
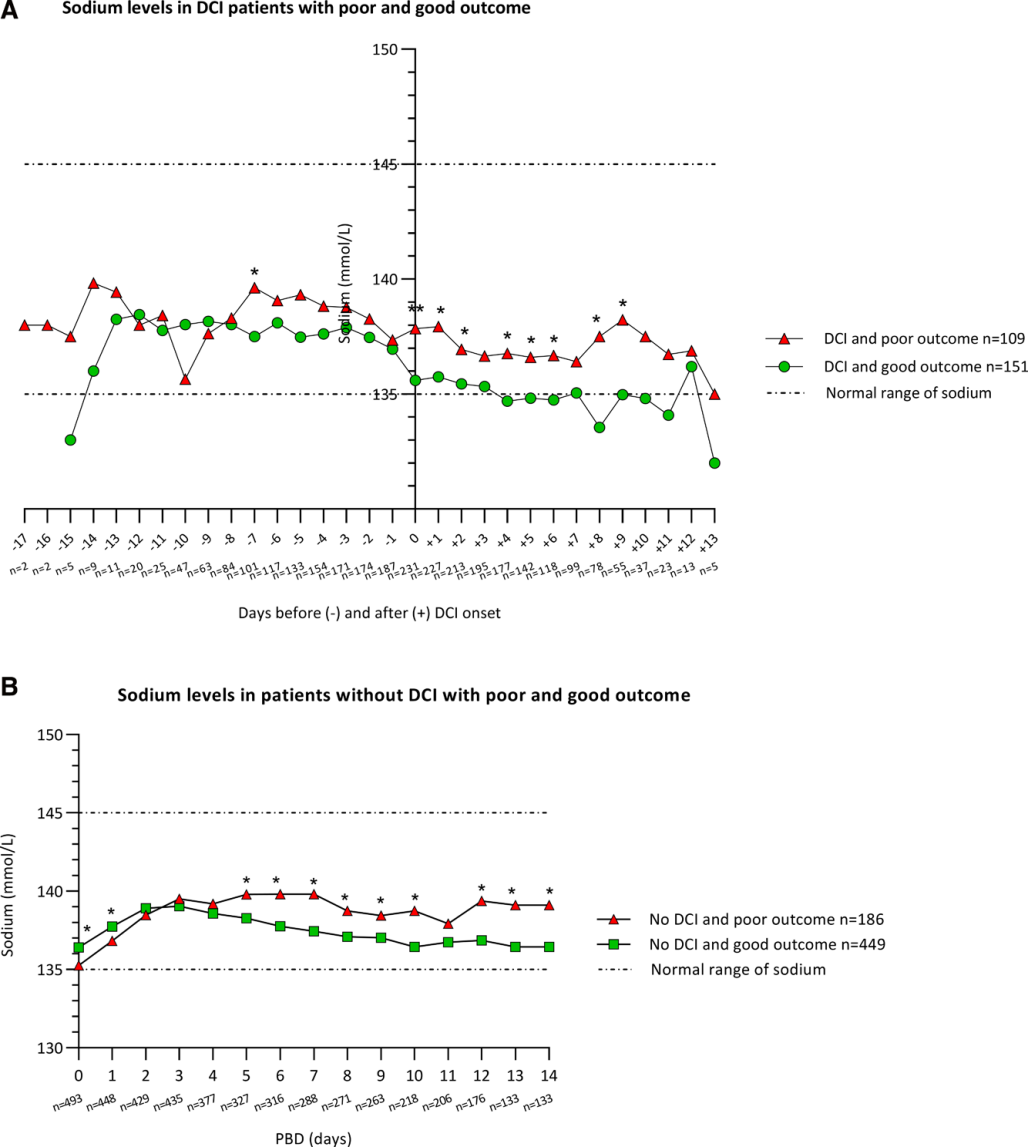
Daily sodium levels in: **A**, delayed cerebral ischemia (DCI) patients with poor and good outcome at 6 months after aneurysmal subarachnoid hemorrhage (aSAH). Days are centered around the day of DCI onset (DCI 0). **B**, Daily sodium levels in patients without DCI with poor and good outcome at 6 months after aSAH. Days are presented as postbleed days (PBD) with PBD 0 as the day of aSAH ictus. The number of available sodium measurements (*n* = *x*) per day are noted under the *x*-axis. *p < 0.05, **p < 0.001.

Within the DCI group, patients with poor outcome had significantly higher sodium levels on days DCI –7, 0, +1, +2, +4, +5, +6, +8, and +9 than patients with good outcome (**Fig. 2*A***). In multivariate logistic regression analyses, higher sodium levels on DCI –7, 0, +1, +2, +4, +5, +8, and +9 days were significantly associated with poor outcome in DCI patients (**Table S7*A***, http://links.lww.com/CCM/H480).

Within the no-DCI group, patients with poor outcome had significantly lower sodium levels on PBD 0 and 1, but significantly higher sodium levels on PBD 5–10 and PBD 12–14 than patients with good outcome (**Fig. 2*B***). In multivariate logistic regression analysis, higher sodium levels on PBD 6–10 and PBD 12–14 were significantly associated with poor outcome in no-DCI patients (**Table S7*B***, http://links.lww.com/CCM/H480).

Within DCI and no-DCI patients, not hyponatremia but hypernatremia (before DCI onset, after DCI onset and during admission) was significantly associated with poor outcome in multivariate logistic regression analysis (**Table S8**, ***A*** and ***B***, http://links.lww.com/CCM/H480).

**Figure 3.**
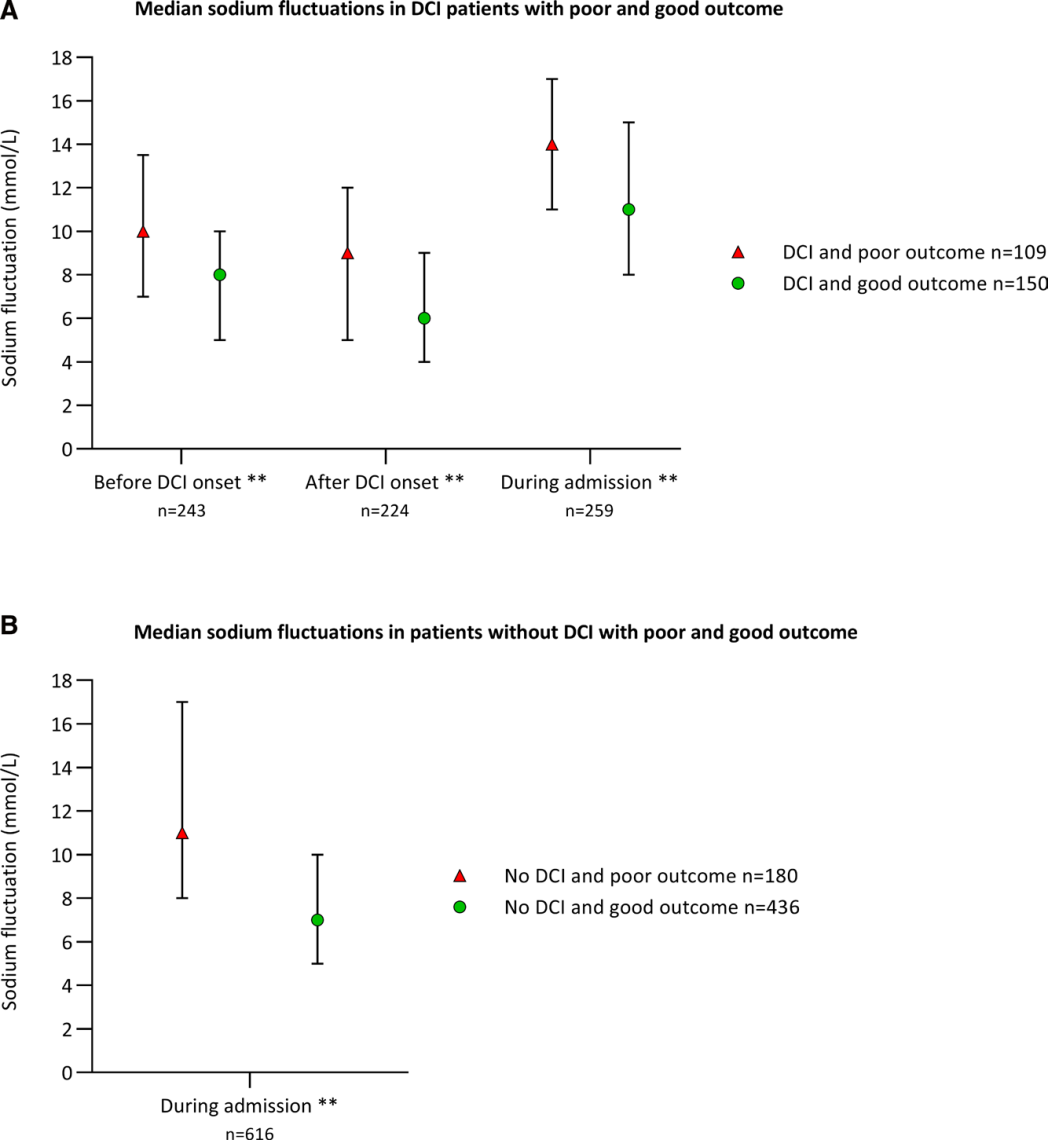
Median (interquartile range) sodium fluctuations **(A)** in delayed cerebral ischemia (DCI) patients with poor and good outcome, and **(B)** in patients without DCI with poor and good outcome. Sodium fluctuations were defined as the difference between the minimum and maximum sodium level in the concerning time interval. The number of available sodium fluctuation (*n* = *x*) per interval are noted under the *x*-axis. *p < 0.05, **p < 0.001.

Within DCI and no-DCI patients, patients with poor outcome had significantly greater sodium fluctuations than patients with good outcome (**Fig. 3**, ***A*** and ***B***). In multivariate logistic regression analysis, greater sodium fluctuations before DCI onset were significantly associated with poor outcome within DCI patients (aOR 1.08; 95% CI, 1.02–1.14; *p* = 0.008), and greater sodium fluctuations during admission were significantly associated with poor outcome within no-DCI patients (aOR 1.11; 95% CI, 1.07–1.16; *p* < 0.001) (Table S8, *A* and *B*, http://links.lww.com/CCM/H480).

## DISCUSSION

This study in a large cohort of aSAH patients shows that sodium levels, hyponatremia and fluctuations of sodium levels are not predictive of the development of DCI. However, higher sodium levels, hypernatremia and greater sodium fluctuations were associated with poor clinical outcome, irrespective of the occurrence of DCI.

Since 1985, the importance of volemic status and dysnatremia has been emphasized as a possible risk factor for DCI (16–18), leading to the recommendation of maintaining euvolemia and preventing/treating hyponatremia to prevent DCI (8, 9, 19). Thus, the use of fludrocortisone or hypertonic saline was deemed reasonable, although evidence is weak (8, 19, 62). Although hyponatremia is one of the most common electrolyte disturbances after aSAH, guidelines vary widely on management of dysnatremia and often lack recommendations on electrolyte monitoring (8, 9, 63–66), perhaps resulting in practice variation across hospitals worldwide. Despite these recommendations, studies still find that hyponatremia occurs more often in DCI patients (34, 35), but with conflicting associations between hyponatremia, sodium fluctuations, and DCI (31, 34, 38, 40–44). Significantly lower sodium levels, within the hyponatremia (30) and normal range (39), and natriuresis were observed more in DCI patients than no-DCI patients (30, 32). Similarly, evidence regarding poor outcome and sodium is conflicting; some find significant associations between poor outcome, hyponatremia (35, 47), hypernatremia (31, 37, 46, 48, 49) or both (50), whereas others do not find any significant associations (34, 42). Furthermore, a recent randomized trial showed that continuous infusion of 20% hypertonic saline for a minimum of 48 hours did not have a significant effect on clinical outcome than standard care after moderate to severe traumatic brain injury (TBI) (67). This is despite the observation that patients in the intervention group had higher blood osmolality and sodium levels than the control group. Hereby suggesting a lack of effect of sodium on clinical outcome. However, only moderate to severe TBI patients were included and sodium levels were measured only the first 5 days, while we demonstrate that sodium levels start to differ around day 4 after aSAH between patients with poor and good outcome. Additionally, the power of the trial was limited, the CIs for the findings were wide and the pathophysiological mechanisms triggered by aneurysmal rupture is essentially different from TBI, which makes it difficult to extrapolate these results to aSAH. Interestingly, recent studies consistently show an association between sodium fluctuations and poor outcome (42, 43, 49, 53), and patients with poor outcome have significantly higher mean sodium levels than good outcome, which is in line with our findings (52, 53). In previous reports, sodium and free water disorders were more often observed in patients with a ruptured ACOM aneurysm; however, we did not find differences between these patients (68). Comparison of different studies is virtually impossible due to heterogeneity in the definition of hyponatremia and sodium fluctuations, methodology, frequency/timing of sampling, without regards to the day of DCI onset and the timing of clinical outcome assessment (11, 68). Similarly, there is significant variation in fluid management across hospitals (21, 69, 70). Of note, it is well known that fluid balance does not reflect volemic status adequately (71) and higher fluid input, besides fluid restriction, is also associated with DCI (20, 22) and poor outcome (21). Vergouw et al (20) even showed that significant reduction of fluid input was possible while maintaining adequate cardiac preload and thus CBF. This suggests that current approaches for assessing and maintaining optimal volemic status in aSAH need to be further optimized.

We show that lower sodium levels, including hyponatremia, hypernatremia, and greater sodium fluctuations are not predictive of the development of DCI. Rather, significantly lower sodium levels are observed in patients after DCI occurs compared to patients without DCI, and the presence of DCI seems to be predictive of developing hyponatremia, lower sodium levels, and smaller sodium fluctuations. This can be partially explained as DCI patients received hypertension induction, which is standard treatment of DCI in our institution. Hypertension can cause natriuresis, resulting in lower sodium levels, while noradrenalin is associated with reduced natriuresis (72). However, enhanced salt loss due to cerebral salt wasting or a more pronounced syndrome of inappropriate antidiuretic hormone (8, 25–29) due to DCI, can further contribute to lower sodium levels. Of note, from our data it is not possible to differentiate the underlying mechanisms of reduced sodium levels. Interestingly, we find that higher sodium levels, hypernatremia, and greater sodium fluctuations (independently) are associated with poor outcome in DCI and no-DCI patients. To the best of our knowledge, this has not been demonstrated before in this manner. Although, sodium levels, including changes, seem to be the result of DCI, our results suggest that changes or any deviation from the physiologic state of sodium levels of the patient could have a negative impact on the clinical outcome. However, the influence of other medication, (excessive) fluid intake or cardiac dysfunction, which could affect the sodium/water balance and volemic status, could not be ruled out except for hypertonic saline use. Since relatively great sodium fluctuations can occur within the clinically accepted normal range of sodium, a single measurement of sodium may fail to capture these relevant changes. Therefore, the focus should perhaps shift to sodium fluctuations, or even osmolality. Minimizing sodium fluctuations to maintain a stable sodium trajectory and osmolality might be of more importance than sodium levels and correcting hyponatremia. To minimize sodium fluctuations, a narrower range of “normal” sodium levels could be considered with the upper limit being lowered as patients with poor outcome had higher sodium levels than patients with good outcome. This should be considered carefully as the management of sodium may have limited influence on outcome due to the multifactorial and not-fully comprehended nature of the pathophysiology triggered by aSAH (10, 73).

Because previous studies have shown that fluid restriction and excessive fluid intake are risk factors for DCI and poor outcome, and our findings suggest that sodium is associated with poor outcome, future research should focus on sodium fluctuations while taking volemic status, underlying etiology of dysnatremia, medication and cardiac function into account (8, 16–18, 74). Monitoring sodium levels more than once a day in patients will provide a more accurate reflection of the sodium trajectory after aSAH. Furthermore, recording fluid balances prospectively may improve its reliability. If the importance of sodium and fluid balance is established, it might create incentive to record fluid balances accurately on the wards despite its labor-intensive nature or new ways to assess this. This study shows a robust signal and could serve as a prequel to future trials with a rigorous preselection of patients targeting the underlying causes of dysnatremia while controlling for confounders such as hypertension or hypotension and DCI. This could fill the gaps in knowledge and future directions (74).

Strengths of our study include the prospective registry use with standardized/structured outcome assessment, large sample size, and taking the day of DCI onset into account. Limitations are the retrospective collection of sodium levels resulting in missing and variation in available sodium levels between patients (ascertainment bias), conducting multiple statistical analyses (type 1 error), an underestimation or overestimation of the occurrence of hyponatremia and imprecise determination of the timing of hyponatremia occurrence (75). Oral/IV fluid intake, medications affecting sodium/water balance, and fluid balances were not included as it is often improperly recorded and unreliable when collected retrospectively (76–80). Lastly, patients who died within 3 days were excluded, which could affect the generalizability of our findings.

## CONCLUSIONS

Hyponatremia and sodium fluctuations still occurred frequently in our aSAH cohort despite our institutional protocol, but was not associated with DCI. The changes in sodium levels observed in DCI patients are likely the result of DCI and its therapeutic management. However, disorders in sodium levels, including mild changes, appear to have a negative impact on clinical outcome at 6 months in aSAH patients with and without DCI.

## Supplementary Material

## References

[1] van Gijn J Kerr RS Rinkel GJE: Subarachnoid haemorrhage. Lancet. 2007; 369:306–318 17258671 10.1016/S0140-6736(07)60153-6

[2] Feigin VL Lawes CM Bennett DA: Worldwide stroke incidence and early case fatality reported in 56 population-based studies: A systematic review. Lancet Neurol. 2009; 8:355–369 19233729 10.1016/S1474-4422(09)70025-0

[3] Nieuwkamp DJ Setz LE Algra A: Changes in case fatality of aneurysmal subarachnoid haemorrhage over time, according to age, sex, and region: A meta-analysis. Lancet Neurol. 2009; 8:635–642 19501022 10.1016/S1474-4422(09)70126-7

[4] Al-Khindi T MacDonald RL Schweizer TA: Cognitive and functional outcome after aneurysmal subarachnoid hemorrhage. Stroke. 2010; 41:e519–e536 20595669 10.1161/STROKEAHA.110.581975

[5] Duan W Pan Y Wang C: Risk factors and clinical impact of delayed cerebral ischemia after aneurysmal subarachnoid hemorrhage: Analysis from the China National Stroke Registry. Neuroepidemiology. 2018; 50:128–136 29529609 10.1159/000487325

[6] Macdonald RL: Delayed neurological deterioration after subarachnoid haemorrhage. Nat Rev Neurol. 2014; 10:44–58 24323051 10.1038/nrneurol.2013.246

[7] Hammer A Ranaie G Erbguth F: Impact of complications and comorbidities on the intensive care length of stay after aneurysmal subarachnoid haemorrhage. Sci Rep. 2020; 10:6228 32277142 10.1038/s41598-020-63298-9 PMC7148333

[8] Connolly ES Rabinstein AA Carhuapoma JR: Guidelines for the management of aneurysmal subarachnoid hemorrhage: A guideline for healthcare professionals from the American Heart Association/American Stroke Association. Stroke. 2012; 43:1711–1737 22556195 10.1161/STR.0b013e3182587839

[9] Steiner T Juvela S Unterberg A; European Stroke Organization: European Stroke Organization guidelines for the management of intracranial aneurysms and subarachnoid haemorrhage. Cerebrovasc Dis. 2013; 35:93–112 23406828 10.1159/000346087

[10] Dodd WS Laurent D Dumont AS: Pathophysiology of delayed cerebral ischemia after subarachnoid hemorrhage: A review. J Am Heart Assoc. 2021; 10:e021845 34325514 10.1161/JAHA.121.021845 PMC8475656

[11] Tjerkstra MA Labib H Coert BA: Laboratory biomarkers of delayed cerebral ischemia following subarachnoid hemorrhage: A systematic review. J Circ Biomark. 2023; 12:17–25 37056917 10.33393/jcb.2023.2502 PMC10087563

[12] Fantini S Sassaroli A Tgavalekos KT: Cerebral blood flow and autoregulation: Current measurement techniques and prospects for noninvasive optical methods. Neurophotonics. 2016; 3:031411 27403447 10.1117/1.NPh.3.3.031411 PMC4914489

[13] Kreimeier U: Pathophysiology of fluid imbalance. Crit Care. 2000; 4:S3–S7 11255592 10.1186/cc968 PMC3226173

[14] Silverman A Petersen NH: Physiology, cerebral autoregulation. 2020 31985976

[15] Dankbaar JW Slooter AJC Rinkel GJE: Effect of different components of triple-H therapy on cerebral perfusion in patients with aneurysmal subarachnoid haemorrhage: A systematic review. Crit Care. 2010; 14:R23 20175912 10.1186/cc8886 PMC2875538

[16] Hasan D Wijdicks EFM Vermeulen M: Hyponatremia is associated with cerebral ischemia in patients with aneurysmal subarachnoid hemorrhage. Ann Neurol. 1990; 27:106–108 2301918 10.1002/ana.410270118

[17] Wijdicks EFM Vermeulen M Hijdra A: Hyponatremia and cerebral infarction in patients with ruptured intracranial aneurysms: Is fluid restriction harmful? Ann Neurol. 1985; 17:137–140 3977297 10.1002/ana.410170206

[18] Wijdicks EFM Vermeulen M ten Haaf JA: Volume depletion and natriuresis in patients with a ruptured intracranial aneurysm. Ann Neurol. 1985; 18:211–216 4037761 10.1002/ana.410180208

[19] Diringer MN Bleck TP Hemphill JC: Critical care management of patients following aneurysmal subarachnoid hemorrhage: Recommendations from the neurocritical care society’s multidisciplinary consensus conference. Neurocrit Care. 2011; 15:211–240 21773873 10.1007/s12028-011-9605-9

[20] Vergouw LJM Egal M Bergmans B: High early fluid input after aneurysmal subarachnoid hemorrhage: Combined report of association with delayed cerebral ischemia and feasibility of cardiac output-guided fluid restriction. J Intensive Care Med. 2020; 35:161–169 28934895 10.1177/0885066617732747 PMC6927070

[21] van der Jagt M: Fluid management of the neurological patient: A concise review. Crit Care. 2016; 20:126 27240859 10.1186/s13054-016-1309-2 PMC4886412

[22] Rass V Gaasch M Kofler M: Fluid intake but not fluid balance is associated with poor outcome in nontraumatic subarachnoid hemorrhage patients. Crit Care Med. 2019; 47:e555–e562 30985447 10.1097/CCM.0000000000003775

[23] Braun MM Barstow CH Pyzocha NJ: Diagnosis and management of sodium disorders: Hyponatremia and hypernatremia. Am Fam Physician. 2015; 91:299–307 25822386

[24] Agrawal V Agarwal M Joshi SR: Hyponatremia and hypernatremia: Disorders of water balance. J Assoc Physicians India. 2008; 56:956–964 19322975

[25] Kao L Al-Lawati Z Vavao J: Prevalence and clinical demographics of cerebral salt wasting in patients with aneurysmal subarachnoid hemorrhage. Pituitary. 2009; 12:347–351 19462244 10.1007/s11102-009-0188-9

[26] Sherlock M O’Sullivan E Agha A: The incidence and pathophysiology of hyponatraemia after subarachnoid haemorrhage. Clin Endocrinol (Oxf). 2006; 64:250–254 16487432 10.1111/j.1365-2265.2006.02432.x

[27] Betjes MGH: Hyponatremia in acute brain disease: The cerebral salt wasting syndrome. 2002; 13:9–14 10.1016/s0953-6205(01)00192-3 11836078

[28] Hannon MJ Behan LA O’Brien MMC: Hyponatremia following mild/moderate subarachnoid hemorrhage is due to SIAD and glucocorticoid deficiency and not cerebral salt wasting. J Clin Endocrinol Metab. 2014; 99:291–298 24248182 10.1210/jc.2013-3032

[29] Suarez JI: Diagnosis and management of subarachnoid hemorrhage. Continuum (Minneap Minn). 2015; 21:1263–1287 26426230 10.1212/CON.0000000000000217

[30] Okuchi K Fujioka M Fujikawa A: Rapid natriuresis and preventive hypervolaemia for symptomatic vasospasm after subarachnoid haemorrhage. Acta Neurochir. 1996; 138:951–6; discussion 956 8890992 10.1007/BF01411284

[31] Qureshi AI Suri MFK Sung GY: Prognostic significance of hypernatremia and hyponatremia among patients with aneurysmal subarachnoid hemorrhage. Neurosurgery. 2002; 50:749–755, discussion 755-756 11904025 10.1097/00006123-200204000-00012

[32] Igarashi T Moro N Katayama Y: Prediction of symptomatic cerebral vasospasm in patients with aneurysmal subarachnoid hemorrhage: Relationship to cerebral salt wasting syndrome. Neurol Res. 2007; 29:835–841 17767804 10.1179/016164107X228624

[33] Nakagawa I Kurokawa S Nakase H: Hyponatremia is predictable in patients with aneurysmal subarachnoid hemorrhage—clinical significance of serum atrial natriuretic peptide. Acta Neurochir. 2010; 152:2147–2155 20680650 10.1007/s00701-010-0735-1

[34] Zheng B Qiu Y Jin H: A predictive value of hyponatremia for poor outcome and cerebral infarction in high-grade aneurysmal subarachnoid haemorrhage patients. J Neurol Neurosurg Psychiatry. 2011; 82:213–217 20667862 10.1136/jnnp.2009.180349

[35] Vrsajkov V Javanović G Stanisavljević S: Clinical and predictive significance of hyponatremia after aneurysmal subarachnoid hemorrhage. Balkan Med J. 2012; 29:243–246 25207008 10.5152/balkanmedj.2012.037 PMC4115839

[36] Maimaitili A Maimaitili M Rexidan A: Pituitary hormone level changes and hyponatremia in aneurysmal subarachnoid hemorrhage. Exp Ther Med. 2013; 5:1657–1662 23837049 10.3892/etm.2013.1068 PMC3702695

[37] Beseoglu K Etminan N Steiger HJ: The relation of early hypernatremia with clinical outcome in patients suffering from aneurysmal subarachnoid hemorrhage. Clin Neurol Neurosurg. 2014; 123:164–168 24956546 10.1016/j.clineuro.2014.05.022

[38] Hendrix P Foreman PM Harrigan MR: Association of Plasminogen Activator Inhibitor 1 (SERPINE1) polymorphisms and aneurysmal subarachnoid hemorrhage. World Neurosurg. 2017; 105:672–677 28599907 10.1016/j.wneu.2017.05.175

[39] Uozumi Y Mizobe T Miyamoto H: Decreased serum sodium levels predict symptomatic vasospasm in patients with subarachnoid hemorrhage. J Clin Neurosci. 2017; 46:118–123 28887070 10.1016/j.jocn.2017.08.037

[40] Quinn L Tian DH Fitzgerald E: The association between hyponatraemia and long-term functional outcome in patients with aneurysmal subarachnoid haemorrhage: A single centre prospective cohort study. J Clin Neurosci. 2020; 78:353–359 32622650 10.1016/j.jocn.2020.06.003

[41] Liu H Xu Q Li A: Nomogram for predicting delayed cerebral ischemia after aneurysmal subarachnoid hemorrhage in the Chinese population. J Stroke Cerebrovasc Dis. 2020; 29:105005 32807421 10.1016/j.jstrokecerebrovasdis.2020.105005

[42] Bales J Cho S Tran TK: The effect of hyponatremia and sodium variability on outcomes in adults with aneurysmal subarachnoid hemorrhage. World Neurosurg. 2016; 96:340–349 27637165 10.1016/j.wneu.2016.09.005

[43] Eagles ME Tso MK Loch Macdonald R: Significance of fluctuations in serum sodium levels following aneurysmal subarachnoid hemorrhage: An exploratory analysis. 2019; 131:420–425 10.3171/2018.3.JNS173068 30117765

[44] Cohen J Delaney A Anstey J: Dysnatremia and 6-month functional outcomes in critically ill patients with aneurysmal subarachnoid hemorrhage: A prospective cohort study. Crit Care Explor. 2021; 3:e0445 34124687 10.1097/CCE.0000000000000445 PMC8189636

[45] Harada T Uozumi Y Fukuoka H; Kobe University SAH study collaborators: The impact of hormonal dynamics and serum sodium fluctuations on symptomatic vasospasm after subarachnoid hemorrhage. J Clin Neurosci. 2022; 103:131–140 35872447 10.1016/j.jocn.2022.07.016

[46] Wartenberg KE Schmidt JM Claassen J: Impact of medical complications on outcome after subarachnoid hemorrhage. Crit Care Med. 2006; 34:617–23; quiz 624 16521258 10.1097/01.ccm.0000201903.46435.35

[47] Saramma P Menon RG Srivastava A: Hyponatremia after aneurysmal subarachnoid hemorrhage: Implications and outcomes. J Neurosci Rural Pract. 2013; 4:24–28 23546343 10.4103/0976-3147.105605 PMC3579037

[48] Alimohamadi M Saghafinia M Alikhani F: Impact of electrolyte imbalances on the outcome of aneurysmal subarachnoid hemorrhage: A prospective study. Asian J Neurosurg. 2016; 11:29–33 26889275 10.4103/1793-5482.154978 PMC4732238

[49] Tam CW Shum HP Yan WW: Impact of dysnatremia and dyskalemia on prognosis in patients with aneurysmal subarachnoid hemorrhage: A retrospective study. Indian J Crit Care Med. 2019; 23:562–567 31988546 10.5005/jp-journals-10071-23292 PMC6970205

[50] Roquer J Cuadrado-Godia E Guimaraens L: Short- and long-term outcome of patients with aneurysmal subarachnoid hemorrhage. Neurology. 2020; 95:e1819–e1829 32796129 10.1212/WNL.0000000000010618 PMC7682825

[51] Kieninger M Kerscher C Brundl E: Acute hyponatremia after aneurysmal subarachnoid hemorrhage: Frequency, treatment, and outcome. J Clin Neurosci. 2021; 88:237–242 33992191 10.1016/j.jocn.2021.04.004

[52] Chua MMJ Enriquez-Marulanda A Gomez-Paz S: Sodium variability and probability of vasospasm in patients with aneurysmal subarachnoid hemorrhage. J Stroke Cerebrovasc Dis. 2022; 31:106186 34749298 10.1016/j.jstrokecerebrovasdis.2021.106186 PMC9000130

[53] Jin D Jin S Liu B: Association between serum sodium and in-hospital mortality among critically ill patients with spontaneous subarachnoid hemorrhage. Front Neurol. 2022; 13:1025808 36388235 10.3389/fneur.2022.1025808 PMC9662614

[54] Report of world federation of neurological surgeons committee on a universal subarachnoid hemorrhage grading scale. J Neurosurg. 1988; 68:985–986 3131498 10.3171/jns.1988.68.6.0985

[55] Frontera JA Claassen J Schmidt JM: Prediction of symptomatic vasospasm after subarachnoid hemorrhage: The modified fisher scale. Neurosurgery. 2006; 59:21–7; discussion 21 16823296 10.1227/01.neu.0000243277.86222.6c

[56] Vergouwen MDI Vermeulen M van Gijn J: Definition of delayed cerebral ischemia after aneurysmal subarachnoid hemorrhage as an outcome event in clinical trials and observational studies: Proposal of a multidisciplinary research group. Stroke. 2010; 41:2391–2395 20798370 10.1161/STROKEAHA.110.589275

[57] Wilson JT Hareendran A Grant M: Improving the assessment of outcomes in stroke: Use of a structured interview to assign grades on the modified Rankin Scale. Stroke. 2002; 33:2243–2246 12215594 10.1161/01.str.0000027437.22450.bd

[58] Wilson JT Hareendran A Hendry A: Reliability of the modified Rankin Scale across multiple raters: Benefits of a structured interview. Stroke. 2005; 36:777–781 15718510 10.1161/01.STR.0000157596.13234.95

[59] Janssen PM Visser NA Dorhout Mees SM: Comparison of telephone and face-to-face assessment of the modified Rankin Scale. Cerebrovasc Dis. 2010; 29:137–139 19955737 10.1159/000262309

[60] Qureshi AI Sung GY Razumovsky AY: Early identification of patients at risk for symptomatic vasospasm after aneurysmal subarachnoid hemorrhage. Crit Care Med. 2000; 28:984–990 10809270 10.1097/00003246-200004000-00012

[61] Germans MR Jaja BNR De Oliviera Manoel AL: Sex differences in delayed cerebral ischemia after subarachnoid hemorrhage. J Neurosurg. 2018; 129:458–464 28862545 10.3171/2017.3.JNS162808

[62] Shah K Turgeon RD Gooderham PA: Prevention and treatment of hyponatremia in patients with subarachnoid hemorrhage: A systematic review. World Neurosurg. 2018; 109:222–229 28987848 10.1016/j.wneu.2017.09.182

[63] Bederson JB Connolly ES Batjer HH; American Heart Association: Guidelines for the management of aneurysmal subarachnoid hemorrhage: A statement for healthcare professionals from a special writing group of the stroke council, American Heart Association. Stroke. 2009; 40:994–1025 19164800 10.1161/STROKEAHA.108.191395

[64] D’Souza S: Aneurysmal subarachnoid hemorrhage. J Neurosurg Anesthesiol. 2015; 27:222–240 25272066 10.1097/ANA.0000000000000130 PMC4463029

[65] Diringer MN: Management of aneurysmal subarachnoid hemorrhage. Crit Care Med. 2009; 37:432–440 19114880 10.1097/CCM.0b013e318195865a PMC2820121

[66] Van Gijn J Rinkel GJE: Subarachnoid haemorrhage: Diagnosis, causes and management. 2001; 124:249–278 10.1093/brain/124.2.249 11157554

[67] Roquilly A Moyer JD Huet O; Atlanrea Study Group and the Société Française d’Anesthésie Réanimation (SFAR) Research Network: Effect of continuous infusion of hypertonic saline vs standard care on 6-month neurological outcomes in patients with traumatic brain injury: The COBI Randomized Clinical Trial. JAMA. 2021; 325:2056–2066 34032829 10.1001/jama.2021.5561 PMC8150692

[68] Mapa B Taylor BES Appelboom G: Impact of hyponatremia on morbidity, mortality, and complications after aneurysmal subarachnoid hemorrhage: A systematic review. 2016; 85:305–314 10.1016/j.wneu.2015.08.054 26361321

[69] Velly LJ Bilotta F Fabregas N; European Neuroanaesthesia and Critical Care Interest Group (ENIG): Anaesthetic and ICU management of aneurysmal subarachnoid haemorrhage: A survey of European practice. Eur J Anaesthesiol. 2015; 32:168–176 25303971 10.1097/EJA.0000000000000163

[70] Meyer R Deem S Yanez ND: Current practices of triple-H prophylaxis and therapy in patients with subarachnoid hemorrhage. Neurocrit Care. 2011; 14:24–36 20838932 10.1007/s12028-010-9437-z

[71] Hoff RG van Dijk GW Algra A: Fluid balance and blood volume measurement after aneurysmal subarachnoid hemorrhage. Neurocrit Care. 2008; 8:391–397 18172784 10.1007/s12028-007-9043-x

[72] Lang CC Rahman AR Balfour DJ: Effect of noradrenaline on renal sodium and water handling in euhydrated and overhydrated man. Clin Sci (Lond). 1993; 85:487–494 8222516 10.1042/cs0850487

[73] Sehba FA Hou J Pluta RM: The importance of early brain injury after subarachnoid hemorrhage. Prog Neurobiol. 2012; 97:14–37 22414893 10.1016/j.pneurobio.2012.02.003 PMC3327829

[74] Busl KM Rabinstein AA: Prevention and correction of dysnatremia after aneurysmal subarachnoid hemorrhage. Neurocrit Care. 2023; 39:70–80 37138158 10.1007/s12028-023-01735-z

[75] Keirse MJ Hanssens M: Control of error in randomized clinical trials. Eur J Obstet Gynecol Reprod Biol. 2000; 92:67–74 10986437 10.1016/s0301-2115(00)00455-3

[76] Perren A Markmann M Merlani G: Fluid balance in critically ill patients should we really rely on it? 2011 21730928

[77] Diacon A Bell J: Investigating the recording and accuracy of fluid balance monitoring in critically ill patients. South African J Crit Care. 2014; 30:55–57

[78] Asfour HI: Fluid balance monitoring accuracy in intensive care units. IOSR J Nurs Health Sci. 2016; 5:53–62

[79] Nazli A Brigham-Chan F Fernandes M: Adequacy of fluid balance chart documentation on wards. Clin Med (Lond). 2016; 16(Suppl 3):s21 27252324 10.7861/clinmedicine.16-3-s21 PMC4989939

[80] Lim SH Lim ML Bakar Aloweni FA: Audit of the appropriateness and accuracy of fluid intake and output monitoring: Experience in a tertiary hospital. Br J Nurs. 2021; 30:660–664 34109822 10.12968/bjon.2021.30.11.660

