# Supplementary material for: Sodium and Its Impact on Outcome After Aneurysmal Subarachnoid Hemorrhage in Patients With and Without Delayed Cerebral Ischemia
Source: Crit Care Med. 2024 Jan 11;52(5):752–63. doi: 10.1097/CCM.0000000000006182 (PMC11008454; doi:10.1097/CCM.0000000000006182)
Supplement: Supplementary file 1 [file ccm-52-0752-s001.docx]

**Supplementary materials**

**Checklist Observational studies**

STROBE Statement page 3-4

**Supplemental methods and materials**

Definitions of collected data page 5

Patient management page 6

**Supplemental results**

Figure S1 Flowchart patient inclusion page 7

Figure S2A-B Sodium fluctuations in patients with and without DCI page 8

Figure S3 Sodium levels in aSAH patients based on WFNS grade on admission page 9

Figure S4 Sodium fluctuations in aSAH patients based on WFNS grade on admission page 10

Figure S5 Sodium levels in aSAH patients based on ruptured aneurysm location page 11

Figure S6 Sodium fluctuations in aSAH patients based on ruptured aneurysm location page 12

Table S1. Baseline characteristics page 13

Table S2A. Sodium levels before DCI onset or during admission after aSAH page 15

(Figure 1A)

Table S2B. Sodium levels after DCI onset or during admission after aSAH page 16

(Figure 1B)

Table S3A. Sodium levels in patients with DCI with poor and good outcome page 17

(Figure 2A)

Table S3B. Sodium levels in patients without DCI with poor and good outcome page 19

(Figure 2B)

Table S4 The relation between sodium levels before DCI onset and DCI after aSAH page 20

Table S5 The relation between DCI and sodium levels after DCI onset after aSAH page 21

Table S6 The relation between DCI, hyponatremia, hypernatremia and page 22

sodium fluctuations after aSAH

Table S7A The relation between sodium levels and poor outcome page 23

within DCI patients after aSAH

Table S7B The relation between sodium levels and poor outcome page 25

within patients without DCI after aSAH

Table S8A The relation between hyponatremia, hypernatremia, sodium fluctuations page 26

and poor outcome within DCI patients after aSAH

Table S8B The relation between hyponatremia, hypernatremia sodium fluctuations page 27

and poor outcome within patients without DCI after aSAH

**STROBE Statement**

Checklist of items that should be included in reports of *cohort studies.*

|  | Item No | Recommendation | Page No |
| --- | --- | --- | --- |
| **Title and abstract** | 1 | (*a*) Indicate the study’s design with a commonly used term in the title or the abstract |  |
|  |  | (*b*) Provide in the abstract an informative and balanced summary of what was done and what was found | Page 1-2 |
| Introduction | | | |
| Background/rationale | 2 | Explain the scientific background and rationale for the investigation being reported | Page 4 |
| Objectives | 3 | State specific objectives, including any prespecified hypotheses | Page 4-5 |
| Methods | | | |
| Study design | 4 | Present key elements of study design early in the paper | Page 6 |
| Setting | 5 | Describe the setting, locations, and relevant dates, including periods of recruitment, exposure, follow-up, and data collection | Page 6-7 |
| Participants | 6 | (*a*) Give the eligibility criteria, and the sources and methods of selection of participants. Describe methods of follow-up |  |
|  |  | (*b*) For matched studies, give matching criteria and number of exposed and unexposed | Page 6-7 |
| Variables | 7 | Clearly define all outcomes, exposures, predictors, potential confounders, and effect modifiers. Give diagnostic criteria, if applicable | Page 6-8 |
| Data sources/ measurement | 8* | For each variable of interest, give sources of data and details of methods of assessment (measurement). Describe comparability of assessment methods if there is more than one group | Page 6-8 |
| Bias | 9 | Describe any efforts to address potential sources of bias | Page 6 |
| Study size | 10 | Explain how the study size was arrived at | Page 6 |
| Quantitative variables | 11 | Explain how quantitative variables were handled in the analyses. If applicable, describe which groupings were chosen and why | Page 6-8 |
| Statistical methods | 12 | (*a*) Describe all statistical methods, including those used to control for confounding | Page 7-8 |
|  |  | (*b*) Describe any methods used to examine subgroups and interactions |  |
|  |  | (*c*) Explain how missing data were addressed |  |
|  |  | (*d*) If applicable, explain how loss to follow-up was addressed |  |
|  |  | (*e*) Describe any sensitivity analyses |  |
| Results | | |  |
| Participants | 13* | (a) Report numbers of individuals at each stage of study—eg numbers potentially eligible, examined for eligibility, confirmed eligible, included in the study, completing follow-up, and analysed |  |
|  |  | (b) Give reasons for non-participation at each stage | Page 9, Table S1, Figure S1 |
|  |  | (c) Consider use of a flow diagram |  |
| Descriptive data | 14* | (a) Give characteristics of study participants (eg demographic, clinical, social) and information on exposures and potential confounders | Page 9, Table S1, Figure S1-S6 |
|  |  | (b) Indicate number of participants with missing data for each variable of interest |  |
|  |  | (c) Summarise follow-up time (eg, average and total amount) |  |
| Outcome data | 15* | Report numbers of outcome events or summary measures over time | Page 9-11, Table S1, Figure 1, Figure S1-S6, |

| Main results | 16 | (*a*) Give unadjusted estimates and, if applicable, confounder-adjusted estimates and their precision (eg, 95% confidence interval). Make clear which confounders were adjusted for and why they were included |  |
| --- | --- | --- | --- |
|  |  | (*b*) Report category boundaries when continuous variables were categorized | Page 9-11, Table 1,  Table S1-S8B |
|  |  | (*c*) If relevant, consider translating estimates of relative risk into absolute risk for a meaningful time period |  |
| Other analyses | 17 | Report other analyses done—eg analyses of subgroups and interactions, and sensitivity analyses | Page 8 |
| Discussion | | | |
| Key results | 18 | Summarise key results with reference to study objectives | Page 12 |
| Limitations | 19 | Discuss limitations of the study, taking into account sources of potential bias or imprecision. Discuss both direction and magnitude of any potential bias | Page 13-15 |
| Interpretation | 20 | Give a cautious overall interpretation of results considering objectives, limitations, multiplicity of analyses, results from similar studies, and other relevant evidence | Page 12-15 |
| Generalisability | 21 | Discuss the generalisability (external validity) of the study results | Page 14-15 |
| Other information | | | |
| Funding | 22 | Give the source of funding and the role of the funders for the present study and, if applicable, for the original study on which the present article is based | Page 16 |

*Give information separately for exposed and unexposed groups.

**Note:** An Explanation and Elaboration article discusses each checklist item and gives methodological background and published examples of transparent reporting. The STROBE checklist is best used in conjunction with this article (freely available on the Web sites of PLoS Medicine at http://www.plosmedicine.org/, Annals of Internal Medicine at http://www.annals.org/, and Epidemiology at http://www.epidem.com/). Information on the STROBE Initiative is available at http://www.strobe-statement.org.

**Definition of collected data**

- The World Federation of Neurosurgical Societies scale (WFNS) was used to assess the clinical severity of aSAH. If patients were sedated and intubated before their arrival in the treatment center, the WFNS grade was based on the neurological examination at the referral hospital. If patients were sedated and intubated before their arrival in the referral hospital, patients were graded as WFNS 5.

- Aneurysm location was dichotomized into the anterior and posterior circulation. The anterior circulation included aneurysms of the internal carotid artery, anterior communicating artery, anterior cerebral artery, middle cerebral artery, posterior communicating artery, ophthalmic artery, and the choroidal artery. The posterior circulation included aneurysms of the basilar artery, posterior cerebral artery, cerebellar superior artery, cerebellar anterior inferior artery, cerebellar posterior inferior artery and the vertebral artery.

- Rebleeding was defined as a new hemorrhage from the causative aneurysm after the initial hemorrhage with confirmation by a plain non-contrast computer tomography (NCCT) or a sudden neurological deterioration with changes in vital parameters and high clinical suspicion, or both.

- Treatment modalities included no treatment, coiling and clipping, coiling in combination with a stent, a flow diverter, web device and parent vessel occlusion.

- DCI was defined according to Vergouwen et al.: “the occurrence of focal neurological impairment or a decrease of ≥2 points on the Glasgow Coma Scale, which is not immediately apparent after aneurysm occlusion and cannot be attributed to other causes” and/or “the presence of cerebral infarction on CT or MR scan, which was not present after early aneurysm occlusion and which cannot be attributed to other causes such as surgical interventions”. [56]

- Hydrocephalus was defined as a gradual onset of deterioration of consciousness measured on the Glasgow Coma Scale (GCS) with enlarged ventricles on NCCT or with elevated intracranial pressure (ICP) measured at drainage of cerebrospinal fluid (CSF) with lumbar puncture or external ventricular drain.

- The diagnosis meningitis was defined as fever with a positive CSF culture.

- A patient was diagnosed with seizures if treatment with antiepileptic drugs was initiated based on clinical symptoms suggestive for seizures, or after electroencephalography confirmation.

- Clinical outcome was assessed at six months by the modified Rankin Scale (mRS) via a standardized and validated interview. These interviews were taken by research nurses who were trained to perform a structured interview to assess the mRS score.[57-59]

**Patient management**

Patients were treated according to our institutional protocol, which is based on (inter)national guidelines.[8, 19] After presentation at the emergency department, patients were either admitted to the brain care unit or the intensive care unit (ICU). Ruptured aneurysms were treated endovascular (preferably) or surgically as early as feasible, preferably within 24 hours after ictus. Patients received oral nimodipine, 60 mg, six times daily for 21 days. If patients had raised ICP or (suspected) hydrocephalus, an external ventricular drain (EVD), or an external lumbar drain (ELD), was placed. Patients suspected of DCI were treated with noradrenalin-induced normovolemic hypertension induction.

If patients were admitted to the brain care unit, routine laboratory controls, including sodium, were measured daily during the first five days. After day five, sodium was measured on indication only. If patients experienced hyponatremia, 5 to 10 grams sodiumchloride per day was added to their enteral intake and sodium levels were checked daily until sodium levels were normalized. Moreover, a minimal fluid intake of 2 liters per day was aimed for, either orally and/or intravenously with sodiumchloride 0.9%.

In the case of severe hyponatremia sodium levels were not allowed to increase more than 0.5 mmol/L per hour and were frequently checked to prevent osmotic demyelination syndrome and cerebral edema. When patients were admitted to the ICU, sodium was measured more than once a day. In order to maintain euvolemia, ICU-patients received infusion with sodiumchloride 0.9% until the year 2012. Afterwards, ICU-patients received balanced crystalloid fluids (plasma-lyte) intravenously as these fluids are more isotonic to human plasma compared to sodiumchloride 0.9%.

**S1. Flowchart patient inclusion**

1,452 SAH patients screened

294 Patients with non-aneurysmal SAH excluded

N=194 aSAH patients excluded

- 126 death within three days

- 1 DCI status unknown

- 12 unknown sampling time (ictus date unknown)

- 55 no sodium level within first two weeks after ictus

1,158 aSAH patients

964 aSAH patients with a sodium level within the first two weeks after ictus

Hyponatremia n=545 (57%)

No hyponatremia n=419 (43%)

DCI n=277 (29%)

Hyponatremia during admission n=214 (77%)

Hyponatremia before DCI onset n=142/265 (54%)

Hyponatremia after DCI onset n=143/241 (59%)

No DCI n=687 (71%)

Hyponatremia during admission n=331 (48%)

**Figure S1.** Flowchart of the inclusion of patients.


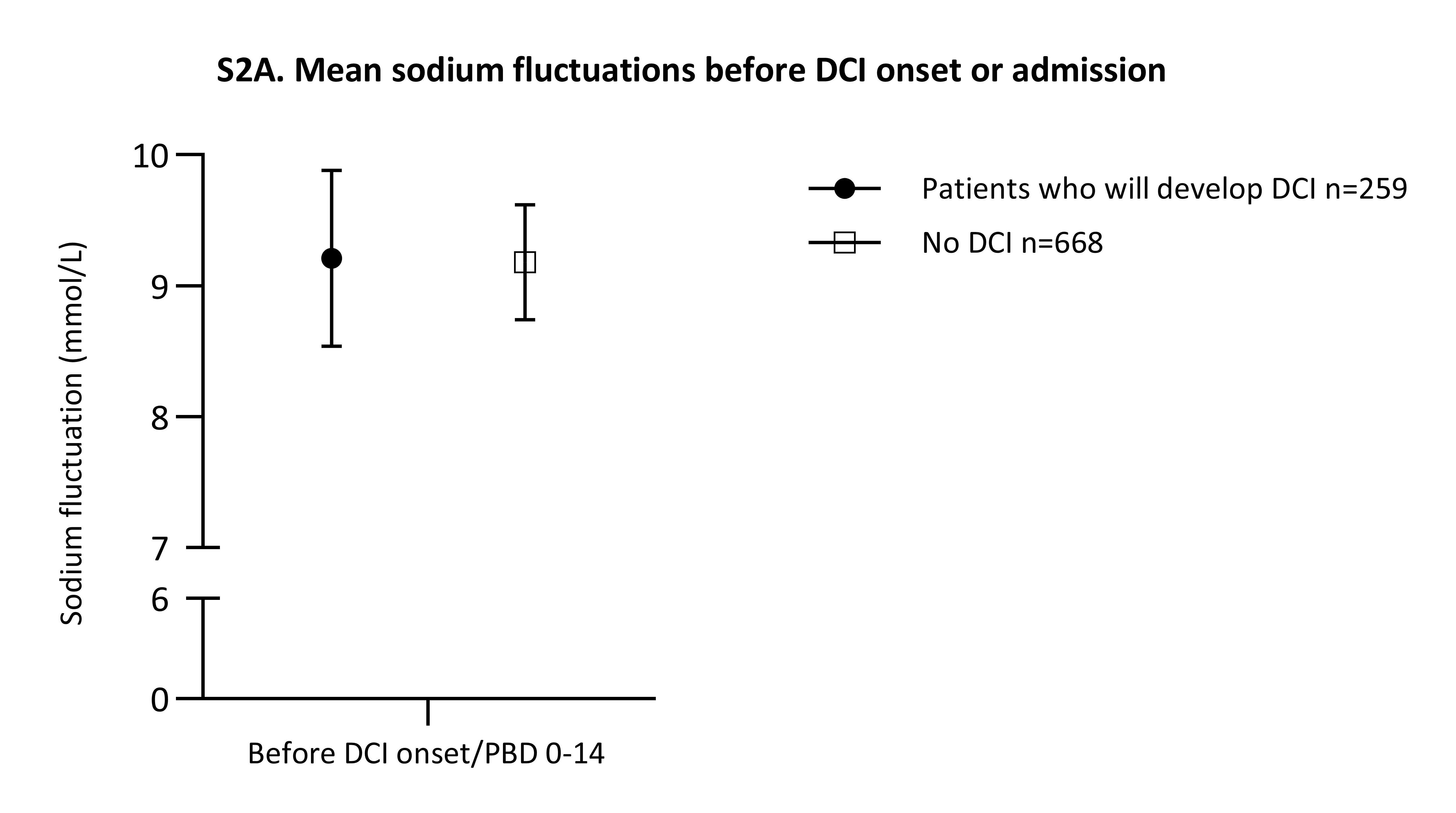

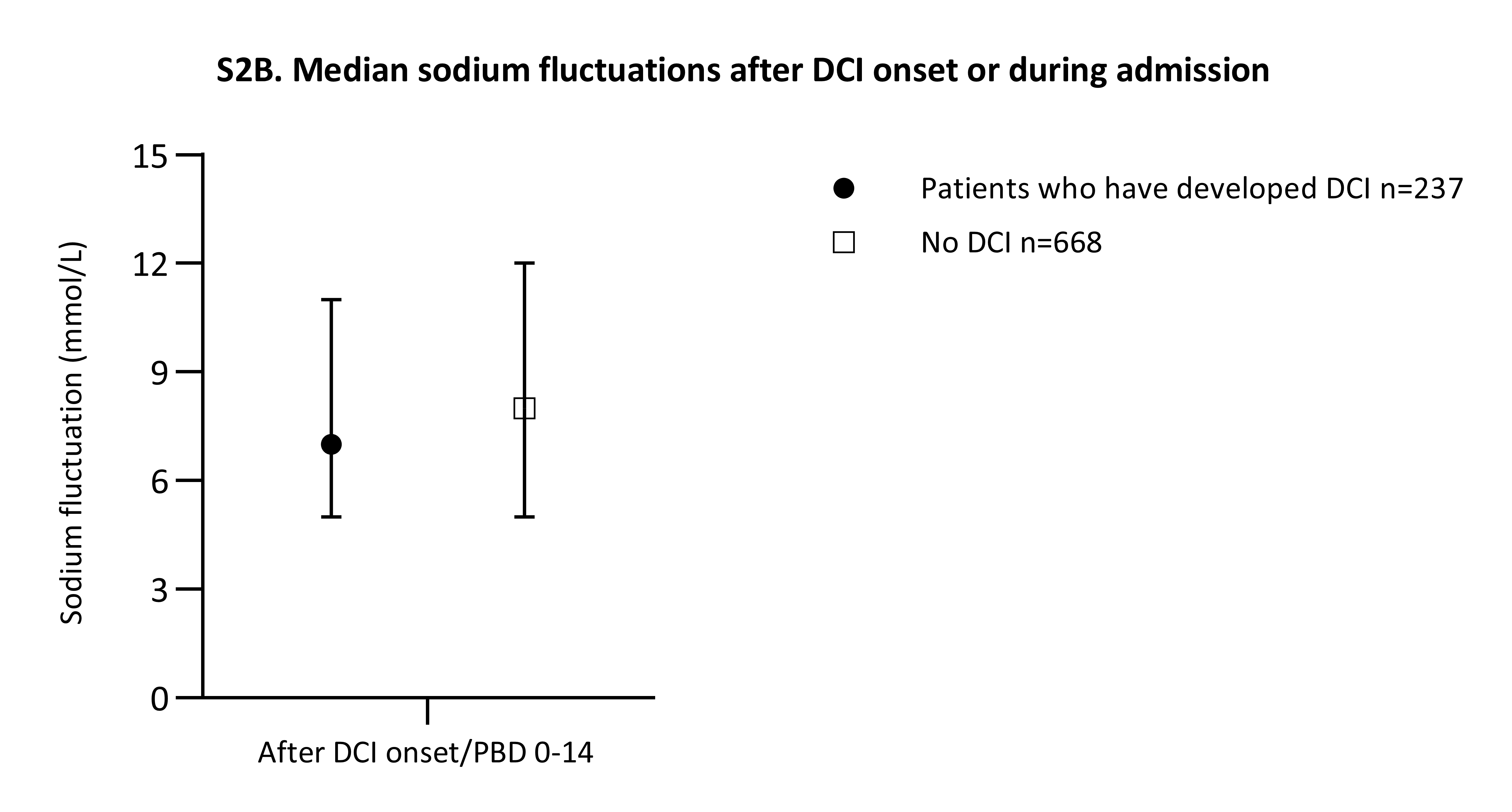


**Figure S2A-B.** Sodium fluctuations in patients with and without DCI. **(A)** Before DCI onset, mean (SD) sodium fluctuations did not differ between patients with and without DCI. **(B)** After DCI onset, median (interquartile range) sodium fluctuations did not differ between patients with and without DCI. Sodium fluctuations were defined as the difference between the minimum and maximum sodium levels before/after the onset of DCI and within 14 days after ictus (PBD 0-14) in patients with DCI and without DCI, respectively. Days are presented as post bleed days (PBD) with PBD 0 as the day of aSAH ictus. Sodium fluctuations could not be calculated for all DCI patients due to having one or less sodium measurements after dividing the time interval into before and after DCI onset and therefore are missing.


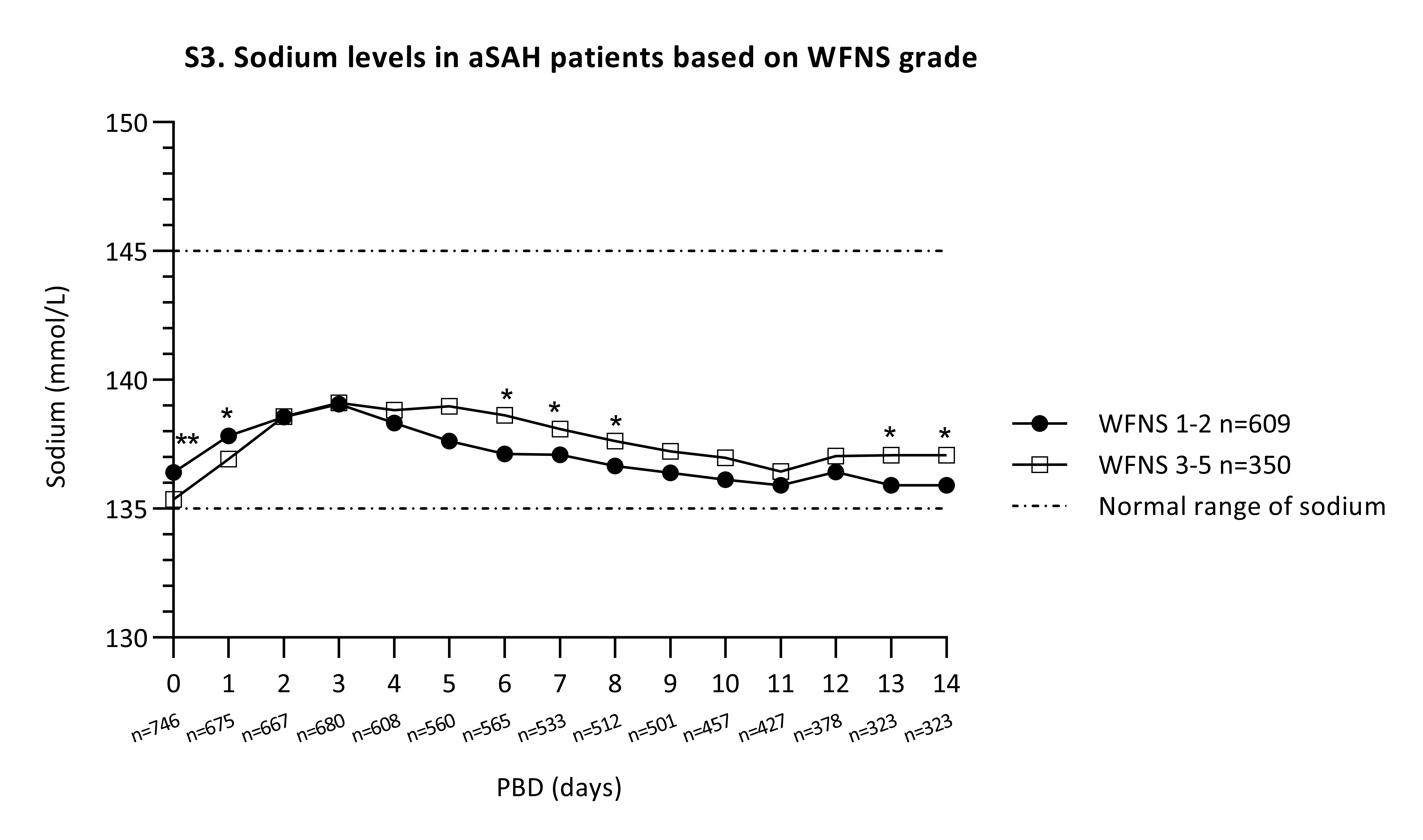


**Figure S3.** Daily sodium levels in WFNS 1-2 and 3-5 patients after aSAH. Days are presented as post bleed days (PBD) with PBD 0 as the day of aSAH ictus. The number of available sodium measurements (n=x) per day are noted under the x-axis. *p<0.05, **p<0.001


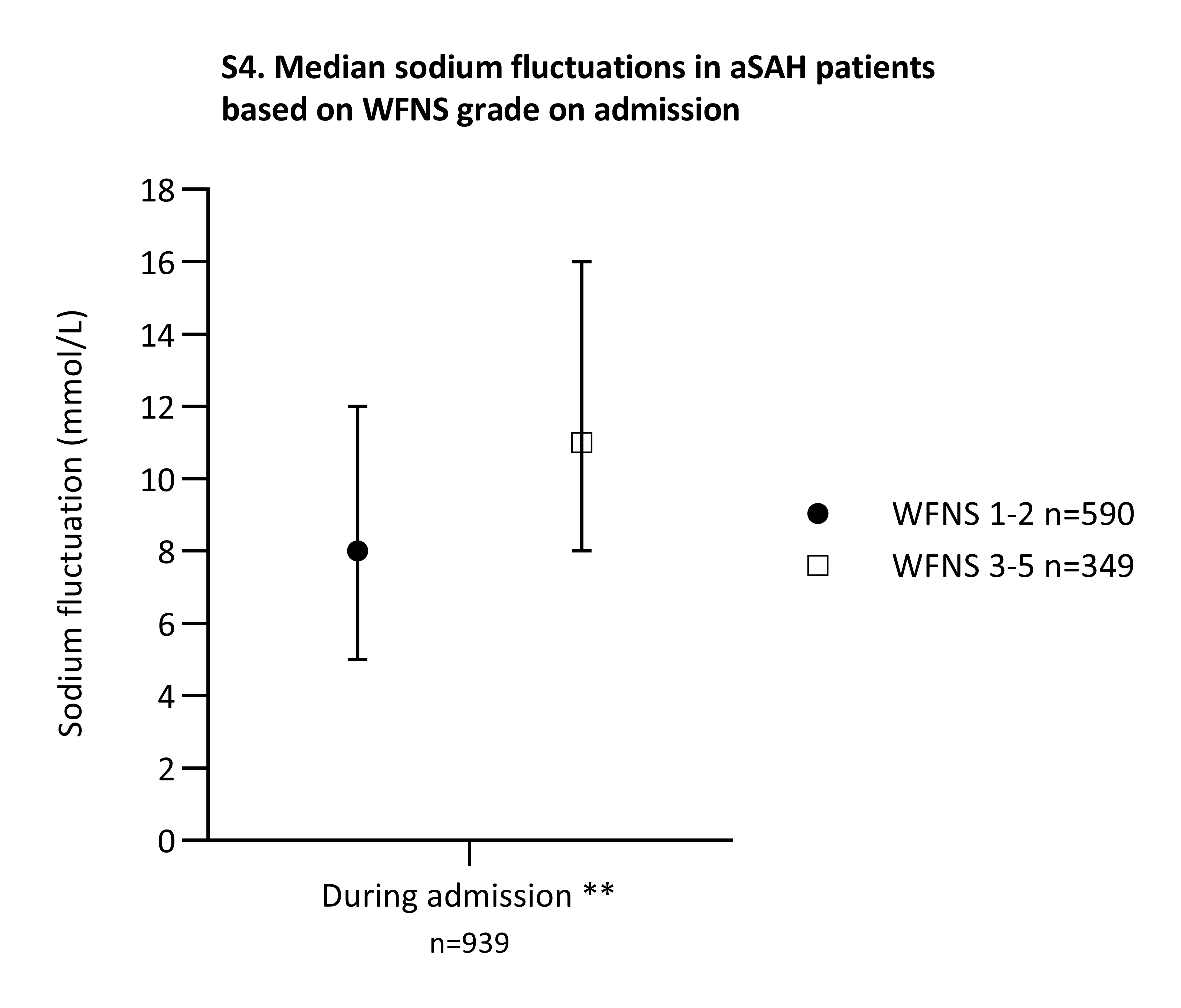


**Figure S4.** Sodium fluctuations in patients in WFNS 1-2 and WFNS 3-5 patients. Sodium fluctuations were defined as the difference between the minimum and maximum sodium level in the concerning time interval. The number of available sodium fluctuation (n=x) are noted under the x-axis. *p<0.05, **p<0.001.


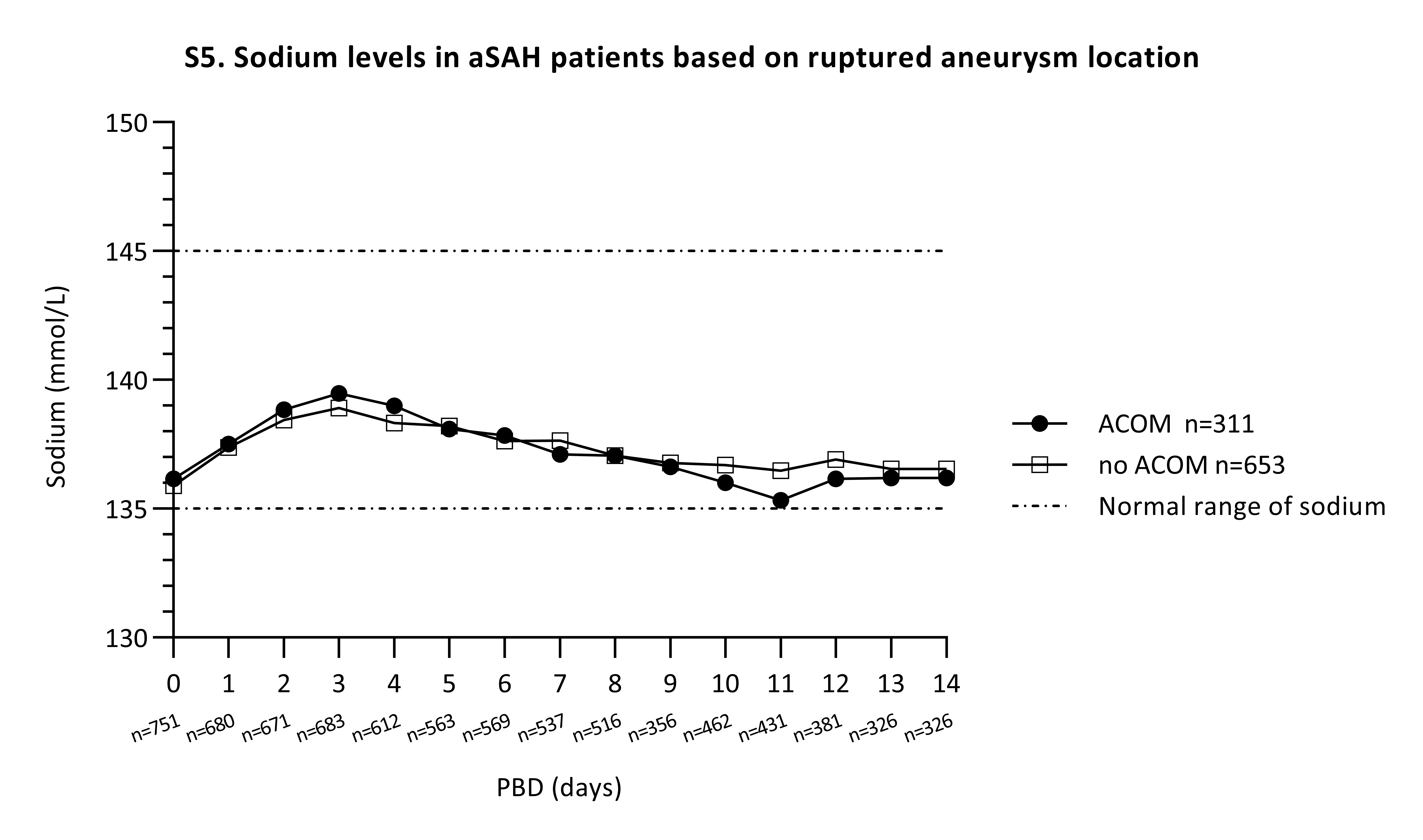


**Figure S5.** Daily sodium levels in patients with and without a ruptured aneurysm of the anterior communicating artery (ACOM). Days are presented as post bleed days (PBD) with PBD 0 as the day of aSAH ictus. The number of available sodium measurements (n=x) per day are noted under the x-axis. *p<0.05, **p<0.001


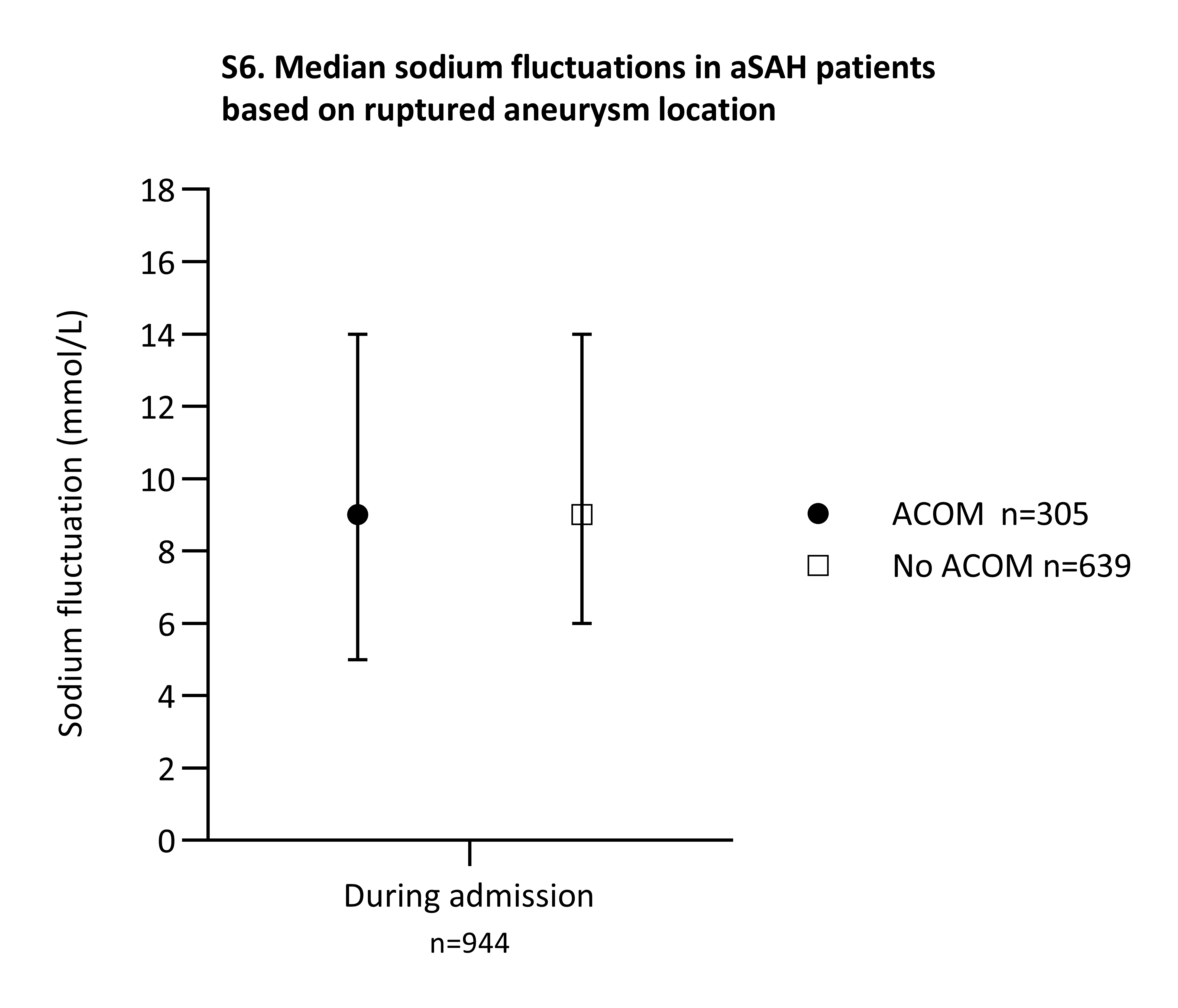


**Figure S6.** Sodium fluctuations during admission in patients with and without a ruptured anterior communicating artery aneurysm (ACOM). Sodium fluctuations were defined as the difference between the minimum and maximum sodium level in the concerning time interval. The number of available sodium fluctuation (n=x) are noted under the x-axis. *p<0.05, **p<0.001.

**Table S1. Baseline characteristics**

|  | **Total aSAH (n=964)** | **DCI (n=277)** | **No DCI (n=687)** | **P-value** |
| --- | --- | --- | --- | --- |
| Age (years), mean (SD) | 57 (13) | 57 (12) | 58 (13) | 0.292 |
| **Female, n (%) *** | 682 (71) | 209 (76) | 473 (69) | **0.041*** |
| Comorbidities, n (%) *^a^*  Hypertension  Hypercholesterolemia  Cardiovascular disease | 317 (33)  129 (13)  158 (16) | 82 (30)  35 (13)  41 (15) | 235 (35)  94 (14)  117 (17) | 0.139  0.665  0.416 |
| WFNS score, n (%) *^a^*  1  2  3  4  5 | 420 (44)  189 (20)  34 (4)  166 (17)  150 (16) | 102 (37)  61 (22)  13 (5)  48 (17)  51 (19) | 318 (46)  128 (19)  21 (3)  118 (17)  99 (14) | 0.067 |
| **Modified Fisher grade, n (%) *^a^* ***  Grade 0  Grade 1  Grade 2  Grade 3  Grade 4 | 29 (3)  54 (6)  27 (3)  268 (28)  585 (61) | 2 (1)  7 (3)  3 (1)  80 (29)  185 (67) | 27 (4)  47 (7)  24 (4)  188 (27)  400 (58) | **<0.001*** |
| Aneurysm location, n (%) *^a^*  Anterior  ACOM  Posterior | 788 (82)  311 (32)  173 (18) | 234 (85)  99 (36)  42 (15) | 554 (81)  99 (36)  131 (19) | 0.154  0.142 |
| **Treatment, n (%) ***  None  Coiling  Clipping  Other | 70 (7)  718 (75)  138 (14)  38 (4) | 6 (2)  213 (77)  50 (18)  8 (3) | 64 (9)  505 (74)  88 (13)  30 (4) | **<0.001*** |
| Complications, n (%) *^a^*  Rebleeding  Clinical  CT-proven  **Hydrocephalus ****  **Meningitis ****  **Seizures **** | 164 (17)  47 (5)  117 (12)  584 (61)  66 (7)  147 (15) | 53 (19)  14 (5)  39 (14)  197 (71)  33 (12)  68 (25) | 111 (16)  33 (5)  78 (11)  387 (56)  33 (5)  79 (12) | 0.266  **<0.001****  **<0.001****  **<0.001**** |
| Hyponatremia, n (%) *^b^*  **PBD 0-14 ****  Prior to DCI onset/PBD 0-14 *^b^*  After DCI onset/PBD 0-14 *^b^* | 545 (57)  473 (49)  474 (49) | 214 (77)  142 (54)  143 (59) | 331 (48)  331 (48)  331 (48) | **<0.001****  0.148  **0.003** |
| Hypernatremia, n (%) *^b^*  **PBD 0-14 ***  Prior to DCI onset/PBD 0-14 *^b^*  After DCI onset/PBD 0-14 *^b^* | 143 (15)  126 (13)  112 (12) | 53 (19)  36 (14)  22 (9) | 90 (13)  90 (13)  90 (13) | **0.021***  0.915  0.109 |
| **Clinical outcome, n (%) *^c^* ****  Good outcome  Poor outcome | 600 (62)  295 (31) | 151 (58)  109 (42) | 449 (70)  186 (30) | **<0.001**** |
| Mortality, n (%) *^c^*  In hospital  Six months’ follow-up | 129 (13)  178 (19) | 44 (16)  62 (24) | 85 (12)  116 (19) | 0.174  0.080 |

*^a^* Information on hypertension (n=24), hypercholesterolemia (n=33), cardiovascular disease (n=24), WFNS grade (n=5), modified Fisher grade (n=1), meningitis (n=1) is missing. Aneurysm location is missing in three patients because an aneurysm in the anterior as well as in the posterior circulation were identified but not clear which aneurysm was the source of the bleeding. ACOM=anterior communicating artery.

*^b^* A total of 265 DCI patients had at least one sodium measurement before DCI onset. Twelve DCI patients had no known sodium levels prior to DCI onset due to missing sodium measurement (n=7) and unknown date of DCI onset (n=5) and therefore unknown sampling time.

A total of 241 DCI patients had at least one sodium measurement after DCI onset. Thirty-six DCI patients had no known sodium levels after DCI onset due to missing sodium measurement after DCI onset (n=31) and unknown date of DCI onset (n=5) and therefore unknown sampling time. A total of 78 DCI patients had an episode of hyponatremia before as well as after DCI onset causing overlap and therefore in a sum of 110%.

*^c^* Clinical outcome at six months was unknown in 69 patients due to lost to lost to follow up (n=22), no further participation (n=10), admittance to hospital (n=1) and unknown reasons (n=12). In the remaining 24 patients, follow-up did not take place at six months but at 12 months after aSAH.

*p<0.05, **p<0.001

**Table S2A. Sodium levels before DCI onset or during admission after aSAH (Figure 1A)**

|  | DCI (n=277) | | No DCI (n=687) | |
| --- | --- | --- | --- | --- |
| **Sodium level on post bleed day (PBD)** | Sodium (mmol/l) *^a^* | N | Sodium (mmol/l) *^a^* | N |
| PBD 0 | 136±3 | 216 | 136±4 | 530 |
| PBD 1 | 137±5 | 183 | 137±4 | 482 |
| PBD 2 | 139±5 | 183 | 139±4 | 462 |
| PBD 3 | 139±5 | 176 | 139±4 | 468 |
| PBD 4 | 138±6 | 140 | 139±4 | 409 |
| PBD 5 | 138±5 | 127 | 139±5 | 352 |
| PBD 6 | 137±5 | 113 | 138±5 | 348 |
| PBD 7 | 137±5 | 96 | 138±5 | 309 |
| PBD 8 | 136±5 | 73 | 138±5 | 292 |
| PBD 9 | 136±5 | 52 | 137±5 | 287 |
| PBD 10 | 136±6 | 33 | 137±5 | 240 |
| PBD 11 | 136±6 | 21 | 137±5 | 223 |
| PBD 12 | 138±6 | 12 | 138±5 | 189 |
| PBD 13 | 137±7 | 8 | 137±5 | 146 |
| PBD 14 | 136±5 | 5 | 137±5 | 146 |

*^a^* Sodium levels are rounded up.

*p<0.05, **p<0.001.

**Table S2B. Sodium levels after DCI onset or during admission after aSAH (Figure 1B)**

|  | DCI (n=277) | | No DCI (n=687) | |
| --- | --- | --- | --- | --- |
| **Sodium level on post bleed day (PBD)** | Sodium (mmol/l) *^a^* | N | Sodium (mmol/l) *^a^* | N |
| PBD 0 | - | 0 | 136±4 | 530 |
| PBD 1 | - | 1 | 137±4 | 482 |
| PBD 2 | 138±3 | 11 | 139±4 | 462 |
| PBD 3 ** | 136±5 | 22 | 139±4 | 468 |
| PBD 4 * | 137±5 | 33 | 139±4 | 409 |
| PBD 5 ** | 136±5 | 59 | 139±5 | 352 |
| PBD 6 * | 137±5 | 76 | 138±5 | 348 |
| PBD 7 ** | 136±5 | 106 | 138±5 | 309 |
| PBD 8 * | 136±5 | 123 | 138±5 | 292 |
| PBD 9 ** | 136±5 | 137 | 137±5 | 287 |
| PBD 10 * | 137±4 | 158 | 137±5 | 240 |
| PBD 11 ** | 135±7 | 171 | 137±5 | 223 |
| PBD 12 ** | 136±4 | 165 | 138±5 | 189 |
| PBD 13 * | 136±5 | 165 | 137±5 | 146 |
| PBD 14 * | 136±5 | 168 | 137±5 | 146 |

*^a^* Sodium levels are rounded up.

*p<0.05, **p<0.001.

**Table S3A. Sodium levels in patients with DCI with poor and good outcome (Figure 2A)**

|  | Poor outcome (n=109) | | Good outcome (n=151) | |
| --- | --- | --- | --- | --- |
| **Sodium level on post bleed day (PBD)** | Sodium (mmol/l) *^a^* | N | Sodium (mmol/l) *^a^* | N |
| DCI -17 | 138±1 | 2 | - | 0 |
| DCI -16 | 138±3 | 2 | - | 0 |
| DCI -15 | 138±3 | 4 | 133 | 1 |
| DCI -14 | 140±4 | 6 | 136±2 | 3 |
| DCI -13 | 139±6 | 7 | 138±3 | 4 |
| DCI -12 | 138±7 | 9 | 139±6 | 11 |
| DCI -11 | 138±5 | 12 | 138±4 | 13 |
| DCI -10 | 136±6 | 17 | 138±5 | 30 |
| DCI -9 | 138±4 | 27 | 138±4 | 36 |
| DCI -8 | 138±5 | 36 | 138±4 | 48 |
| DCI -7 * | 140±5 | 41 | 138±4 | 60 |
| DCI -6 | 139±5 | 50 | 138±4 | 67 |
| DCI -5 | 139±5 | 56 | 137±7 | 77 |
| DCI -4 | 139±5 | 72 | 138±5 | 82 |
| DCI -3 | 139±5 | 78 | 138±5 | 93 |
| DCI -2 | 138±5 | 82 | 137±4 | 92 |
| DCI -1 | 138±7 | 84 | 137±5 | 103 |
| DCI 0 ** | 138±6 | 92 | 136±4 | 139 |
| DCI +1 * | 138±6 | 91 | 136±4 | 136 |
| DCI +2 * | 137±5 | 85 | 135±4 | 128 |
| DCI +3 * | 137±5 | 78 | 135±4 | 117 |
| DCI +4 * | 137±6 | 70 | 135±4 | 107 |
| DCI +5 * | 137±5 | 58 | 135±4 | 84 |
| DCI +6 * | 137±5 | 47 | 135±4 | 71 |
| DCI +7 | 136±5 | 39 | 135±4 | 60 |
| DCI +8 * | 138±5 | 34 | 134±10 | 44 |
| DCI +9 * | 138±6 | 22 | 135±4 | 33 |
| DCI +10 | 138±6 | 16 | 135±5 | 21 |
| DCI +11 | 137±5 | 11 | 134±5 | 12 |
| DCI +12 | 137±7 | 8 | 136±8 | 5 |
| DCI +13 | 135±5 | 3 | 132±7 | 2 |

*^a^* Sodium levels are rounded up.

*p<0.05, **p<0.001.

**Table S3B. Sodium levels in patients without DCI with poor and good outcome (Figure 2B)**

|  | Poor outcome (n=186) | | Good outcome (n=449) | |
| --- | --- | --- | --- | --- |
| **Sodium level on post bleed day (PBD)** | Sodium (mmol/l) *^a^* | N | Sodium (mmol/l) *^a^* | N |
| PBD 0 * | 135±1 | 167 | 136±4 | 326 |
| PBD 1 * | 137±4 | 165 | 138±4 | 283 |
| PBD 2 | 138±5 | 151 | 139±4 | 278 |
| PBD 3 | 140±5 | 130 | 139±4 | 305 |
| PBD 4 | 139±6 | 116 | 139±4 | 261 |
| PBD 5 * | 140±6 | 112 | 138±4 | 215 |
| PBD 6 * | 140±6 | 100 | 138±4 | 216 |
| PBD 7 * | 140±7 | 96 | 137±4 | 192 |
| PBD 8 * | 139±6 | 93 | 137±4 | 178 |
| PBD 9 * | 138±6 | 82 | 137±4 | 181 |
| PBD 10 * | 139±6 | 70 | 136±4 | 148 |
| PBD 11 | 138±6 | 69 | 137±4 | 137 |
| PBD 12 * | 139±6 | 68 | 137±4 | 108 |
| PBD 13 * | 139±6 | 56 | 136±4 | 77 |
| PBD 14 * | 139±6 | 56 | 136±4 | 77 |

*^a^* Sodium levels are rounded up.

*p<0.05, **p<0.001.

**Table S4. The relation between sodium levels before DCI onset and DCI after aSAH**

|  | **DCI *^a^*** | |
| --- | --- | --- |
| **Sodium level on post bleed day (PBD) *^b^*** | OR (95% CI) | aOR (95% CI) *^c^* |
| PBD 0 (n=746) | 0.99 (0.95-1.04) | 1.00 (0.95-1.04) |
| PBD 1 (n=665) | 1.00 (0.96-1.04) | 1.01 (0.97-1.05) |
| PBD 2 (n=645) | 1.00 (0.96-1.04) | 1.00 (0.97-1.04) |
| PBD 3 (n=644) | 1.02 (0.98-1.06) | 1.02 (0.98-1.06) |
| PBD 4 (n=549) | 0.98 (0.95-1.02) | 0.98 (0.95-1.02) |
| PBD 5 (n=479) | 0.97 (0.93-1.01) | 0.97 (0.93-1.01) |
| PBD 6 (n=461) | 0.96 (0.92-1.01) | 0.96 (0.92-1.00) |
| PBD 7 (n=405) | 0.97 (0.93-1.02) | 0.97 (0.93-1.02) |
| PBD 8 (n=365) | 0.96 (0.91-1.01) | 0.96 (0.92-1.02) |
| PBD 9 (n=339) | 0.96 (0.91-1.02) | 0.96 (0.90-1.02) |
| PBD 10 (n=273) | 0.94 (0.88-1.02) | 0.93 (0.86-1.01) |
| PBD 11 (n=244) | 0.96 (0.87-1.06) | 0.96 (0.86-1.06) |
| PBD 12 (n=201) | 1.02 (0.91-1.15) | 1.02 (0.91-1.15) |
| PBD 13 (n=154) | 0.98 (0.86-1.12) | 0.98 (0.85-1.12) |
| PBD 14 (n=151) | 0.95 (0.80-1.13) | 0.94 (0.78-1.13) |

*^a^* 216 (29%), 183 (28%), 183 (28%), 176 (27%), 140 (26%), 127 (27%), 113 (25%), 96 (24%), 73 (20%), 52 (15%), 33 (12%), 21 (9%), 12 (6%), 8 (5%) and 6 (3%) of the patients with a sodium level on PBD 0 to PBD 14 were patients who eventually developed DCI.

*^b^* In patients with DCI, sodium levels before the onset of DCI were included in the analyses. For example, if a patient developed DCI on PBD 4, he was only included for the analysis of sodium levels on PBD 0, 1, 2 and 3.

*^c^* Adjusted for age, sex, WFNS and modified Fisher grade. In the multivariate logistic regression six patients were excluded due to unknown WFNS grade (n=5) and unknown modified Fisher grade (n=1).

*p<0.05, **p<0.001.

**Table S5. The relation between DCI and sodium levels after DCI onset after aSAH**

|  | **The presence of DCI** *^a^* | |
| --- | --- | --- |
| **Variable** | Beta (95% CI) | aBeta (95% CI) *^b^* |
| PBD 0 (n=530) | - | - |
| PBD 1 (n=483) | -0.44 (-8.30 - 7.42) | -1.65 (-9.40 - 6.11) |
| PBD 2 (n=490) | -0.81 (-3.36 - 1.75) | -0.67 (-3.22 - 1.89) |
| PBD 3 (n=442) | **-3.36 (-5.27- - 1.45)**** | **-3.31 (-5.23- -1.38)**** |
| PBD 4 (n=411) | **-2.09 (-3.66 - -0.51)*** | **-2.03 (-3.62- -0.44)*** |
| PBD 5 (n=424) | **-2.37 (-3.72 - -1.03)**** | **-2.34 (-3.70 - -0.98)**** |
| PBD 6 (n=461) | **-1.80 (-3.05 - -0.56)*** | **-1.92 (-3.19 - -0.65)*** |
| PBD 7 (n=415) | **-2.52 (-3.64 - -1.40)**** | **-2.49 (-3.63 - -1.35)**** |
| PBD 8 (n=415) | **-1.69 (-2.72 - -0.65)*** | **-1.55 (-2.62 - -0.49)*** |
| PBD 9 (n=424) | **-1.66 (-2.64 - -0.69)**** | **-1.63 (-2.62 - -0.64)*** |
| PBD 10 (n=398) | **-1.52 (-2.45 - -0.60)*** | **-1.46 (-2.39 - -0.53)*** |
| PBD 11 (n=394) | **-2.10 (-3.24 - -0.96)**** | **-2.02 (-3.19 - -0.86)**** |
| PBD 12 (n=354) | **-2.14 (-3.11 - -1.17)**** | **-2.15 (-3.16 - -1.15)**** |
| PBD 13 (n=311) | **-1.77 (-2.89 - -0.64)*** | **-1.92 (-3.08 - -0.77)*** |
| PBD 14 (n=314) | **-1.75 (-2.87 - -0.64)*** | **-1.90 (-3.05 - -0.76)*** |

*^a^* Univariate linear regression analysis with ***DCI as the independent*** variable and sodium levels as the dependent variable. In patients with DCI, sodium levels after the onset of DCI were included in the analyses. For example, if a patient developed DCI on PBD 4, he was only included for the analysis of sodium levels on PBD 5, 6, 7 and so forth.

*^b^* Adjusted for age, sex, WFNS and modified Fisher grade. In the multivariate linear regression six patients were excluded due to unknown WFNS grade (n=5) and unknown modified Fisher grade (n=1).

*p<0.05, **p<0.001.

**Table S6. The relation between DCI, hyponatremia, hypernatremia and sodium fluctuations after aSAH**

|  | **Hyponatremia *^a^*** | | **Hypernatremia *^b^*** | | **Sodium fluctuations *^c^*** | |
| --- | --- | --- | --- | --- | --- | --- |
| **Variable** | OR (95% CI) | aOR (95% CI) *^d^* | OR (95% CI) | aOR (95% CI) ^d^ | B (95% CI) | aB (95% CI) *^d^* |
| The presence of DCI | **1.57 (1.17-2.11)*** | **1.46 (1.07-1.98)*** | 0.67 (0.41-1.09) | 0.67 (0.41-1.13) | -0.88 (-1.77-0.01) | **-1.25 (-2.11 – -0.39) *** |

*^a^* Univariate logistic regression analysis with ***DCI as the independent*** variable. This analysis was carried out in 928 patients of whom 474 (51%) had hyponatremia after DCI onset or during admission. Patients with DCI were scored as hyponatremia if they had a sodium level <135mmol/L after the day of DCI onset. Patients without DCI were scored as hyponatremia if they had a sodium level <135mmol/L during PBD 0-14.

*^b^* Univariate logistic regression analysis with ***DCI as the independent*** variable. This analysis was carried out in 928 patients of whom 112 (12%) had hypernatremia after DCI onset or during admission. Patients with DCI were scored as hypernatremia if they had a sodium level >145mmol/L after the day of DCI onset. Patients without DCI were scored as hypernatremia if they had a sodium level >145mmol/L during PBD 0-14.

*^c^* Univariate linear regression analysis with ***DCI as the independent*** variable. This analysis was carried out in 905 patients. In patients with DCI, sodium fluctuations were defined as the difference between the highest and lowest sodium value after the onset of DCI. In patients without DCI, sodium fluctuations was defined as the difference between the highest and lowest sodium level during PBD 0-14.

*^d^* Adjusted for age, sex, WFNS and modified Fisher grade. In the multivariate logistic and linear regression, six patients were excluded due to unknown WFNS grade (n=5) and modified Fisher grade (n=1).

*p<0.05, **p<0.001.

**Table S7A. The relation between sodium levels and poor outcome within DCI patients after aSAH**

|  | **Poor outcome within DCI patients** | |
| --- | --- | --- |
| **Sodium level on post bleed day (PBD)** | OR (95% CI) | aOR (95% CI) *^a^* |
| DCI -17 (n=2) | - | - |
| DCI -16 (n=2) | - | - |
| DCI -15 (n=5) | 9.61*10^-14^ (0.00-.) | - |
| DCI -14 (n=9) | 1.40 (0.84-2.34) | 9.53*10^-3^ (0.00-.) |
| DCI -13 (n=11) | 1.05 (0.81-1.39) | 1.23 (0.67-2.26) |
| DCI -12 (n=20) | 0.99 (0.85-1.14) | 0.88 (0.70-1.10) |
| DCI -11 (n=25) | 1.04 (0.86-1.27) | 1.04 (0.85-1.28) |
| DCI -10 (n=47) | 0.91 (0.81-1.04) | 0.92 (0.80-1.05) |
| DCI -9 (n=63) | 0.97 (0.86-1.10) | 0.97 (0.85-1.10) |
| DCI -8 (n=84) | 1.02 (0.91-1.14) | 0.98 (0.87-1.11) |
| **DCI -7 (n=101)** | **1.12 (1.01-1.23)*** | **1.12 (1.01-1.24)*** |
| DCI -6 (n=117) | 1.05 (0.97-1.15) | 1.05 (0.95-1.15) |
| DCI -5 (n=133) | 1.06 (0.99-1.13) | 1.06 (0.98-1.14) |
| DCI -4 (n=154) | 1.05 (0.99-1.12) | 1.06 (0.99-1.15) |
| DCI -3 (n=171) | 1.03 (0.97-1.10) | 1.04 (0.97-1.11) |
| DCI -2 (n=174) | 1.04 (0.97-1.11) | 1.04 (0.96-1.11) |
| DCI -1 (n=187) | 1.01 (0.96-1.06) | 1.01 (0.96-1.06) |
| **DCI 0 (n=231)** | **1.11 (1.04-1.17)**** | **1.09 (1.02-1.16)*** |
| **DCI +1 (n=227)** | **1.10 (1.04-1.17)*** | **1.10 (1.03-1.17)*** |
| **DCI +2 (n=213)** | **1.07 (1.01-1.14)*** | **1.08 (1.01-1.15)*** |
| DCI +3 (n=195) | 1.06 (1.00-1.13) | 1.05 (0.98-1.13) |
| **DCI +4 (n=177)** | **1.10 (1.02-1.18)*** | **1.09 (1.01-1.17)*** |
| **DCI +5 (n=142)** | **1.11 (1.02-1.21)*** | **1.09 (1.00-1.20)*** |
| DCI +6 (n=118) | **1.10 (1.01-1.20)*** | 1.07 (0.97-1.17) |
| DCI +7 (n=99) | 1.08 (0.98-1.19) | 1.08 (0.97-1.20) |
| **DCI +8 (n=78)** | **1.13 (1.02-1.25)*** | **1.21 (1.05-1.38)*** |
| **DCI +9 (n=55)** | **1.12 (1.01-1.26)*** | **1.17 (1.02-1.36)*** |
| DCI +10 (n=37) | 1.10 (0.97-1.26) | 1.15 (0.97-1.36) |
| DCI +11 (n=23) | 1.12 (0.93-1.35) | 1.22 (0.93-1.60) |
| DCI +12 (n=13) | 1.02 (0.86-1.20) | 4.83 (0.00- .) |
| DCI +13 (n=5) | 1.17 (0.76-1.80) | - |

*^a^* Adjusted for age, sex, WFNS and modified Fisher grade. In the multivariate logistic regression, 1, 1, 1, 2, 2, 1, 1, 2, 2, 2, 2, 2, 2, 2, 2, 1, and 1 patients were excluded on DCI -7 to DCI +9, respectively, due to unknown WFNS grade.

*p<0.05, **p<0.001.

**Table S7B. The relation between sodium levels and poor outcome within patients without DCI after aSAH**

|  | **Poor outcome within no DCI patients** | |
| --- | --- | --- |
| **Sodium level on post bleed day (PBD)** | OR (95% CI) | aOR (95% CI) *^a^* |
| PBD 0 (n=493) | **0.92 (0.87-0.97)*** | 1.00 (0.91-1.09) |
| PBD 1 (n=448) | **0.94 (0.90-0.99)*** | 0.97 (0.92-1.03) |
| PBD 2 (n=429) | 0.97 (0.93-1.02) | 1.00 (0.94-1.05) |
| PBD 3 (n=435) | 1.03 (0.98-1.07) | 1.03 (0.98-1.09) |
| PBD 4 (n=377) | 1.03 (0.98-1.09) | 1.03 (0.97-1.09) |
| PBD 5 (n=327) | **1.07 (1.02-1.13)*** | 1.06 (1.00-1.13) |
| PBD 6 (n=316) | **1.10 (1.04-1.16)**** | **1.09 (1.02-1.15)*** |
| PBD 7 (n=288) | **1.10 (1.05-1.17)**** | **1.09 (1.03-1.17)*** |
| PBD 8 (n=271) | **1.08 (1.02-1.14)*** | **1.07 (1.00-1.14)*** |
| PBD 9 (n=263) | **1.07 (1.01-1.14)*** | **1.09 (1.01-1.16)*** |
| PBD 10 (n=218) | **1.11 (1.04-1.19)*** | **1.13 (1.05-1.22)*** |
| PBD 11 (n=206) | 1.05 (0.99-1.12) | 1.06 (0.99-1.14) |
| PBD 12 (n=176) | **1.12 (1.05-1.20)*** | **1.12 (1.04-1.21)*** |
| PBD 13 (n=133) | **1.12 (1.10-1.18)*** | **1.10 (1.01-1.18)*** |
| PBD 14 (n=133) | **1.10 (1.03-1.18)*** | **1.10 (1.01-1.18)*** |

*^a^* Adjusted for age, sex, WFNS and modified Fisher grade. In the multivariate logistic regression, sodium levels of 4, 4, 4, 3, 3, 1, 2, 2, 3, 3, 3, 3, 1, 1 and 1 patients on PBD 0 to 14 were excluded due to unknown WFNS grade.

*p<0.05, **p<0.001.

**Table S8A. The relation between hyponatremia, hypernatremia sodium fluctuations and poor outcome within DCI patients after aSAH**

|  | **Poor outcome** | |
| --- | --- | --- |
| **Post bleed day (PBD)** | OR (95% CI) | aOR (95% CI) *^a^* |
| *Hyponatremia ^b^* |  | |
| Before DCI onset | 1.25 (0.76-2.01) | 1.40 (0.80-2.46) |
| After DCI onset | 0.67 (0.39-1.16) | 0.64 (0.36-1.15) |
| PBD 0-14 | 0.68 (0.38-1.21) | 0.77 (0.41-1.46) |
| *Hypernatremia ^c^* |  | |
| Before DCI onset | **3.42 (1.58-7.37)*** | **2.70 (1.17-6.21)*** |
| After DCI onset | **11.57 (3.30-40.63)**** | **9.27 (2.51-34.21)**** |
| PBD 0-14 | **5.03 (2.55-9.91)**** | **4.16 (2.01-8.62)**** |
| *Sodium fluctuations ^d^* |  | |
| Before DCI onset | **1.08 (1.03-1.15)*** | **1.08 (1.02-1.14)*** |
| After DCI onset | 1.04 (0.99-1.10) | 1.03 (0.98-1.08) |
| PBD 0-14 | **1.05 (1.01-1.10)*** | 1.04 (0.99-1.08) |

*^a^* Adjusted for age, sex, WFNS and modified Fisher grade. In the multivariate logistic regression, two patients were excluded due to unknown WFNS grade.

*^b^* Univariate logistic regression analyses with hyponatremia as the independent variable. These analyses were carried out in 250, 226 and 260 patients on before DCI onset, after DCI onset and PBD 0-14, respectively.

*^c^* Univariate logistic regression analyses with hypernatremia as the independent variable. These analyses were carried out in 250, 226 and 260 patients on before DCI onset, after DCI onset and PBD 0-14, respectively.

*^d^* Univariate logistic regression analyses with sodium fluctuations as the independent variable. These analyses were carried out for 244, 224 and 259 patients on before DCI onset, after DCI onset and PBD 0-14, respectively.

*p<0.05, **p<0.001

**Table S8B. The relation between hyponatremia, hypernatremia sodium fluctuations and poor outcome within patients without DCI after aSAH**

|  | **Poor outcome** | |
| --- | --- | --- |
| **Post bleed day (PBD)** | OR (95% CI) | aOR (95% CI) *^a^* |
| *Hyponatremia ^b^* |  | |
| PBD 0-14 | **2.04 (1.44-2.88)**** | 1.46 (0.94-2.27) |
| *Hypernatremia ^c^* |  | |
| PBD 0-14 | **7.19 (4.34-11.90)**** | **3.84 (2.05-7.19)**** |
| *Sodium fluctuations^d^* |  | |
| PBD 0-14 | **1.16 (1.12-1.20)**** | **1.11 (1.07-1.16)**** |

*^a^* Adjusted for age, sex, WFNS and modified Fisher grade. In the multivariate logistic regression, 4 patients were excluded from PBD 0-14 due to unknown WFNS grade.

*^b^* Univariate logistic regression analyses with hyponatremia as the independent variable. This analysis was carried out in 635 during PBD 0-14.

*^c^* Univariate logistic regression analyses with hypernatremia as the independent variable. This analysis was carried out in 635 during PBD 0-14.

*^d^* Univariate logistic regression analyses with sodium fluctuations as the independent variable. This analysis was carried out for 616 during PBD 0-14.

*p<0.05, **p<0.001.
